# Semi-3D cultures using Laminin 221 as a coating material for human induced pluripotent stem cells

**DOI:** 10.1093/rb/rbac060

**Published:** 2022-09-05

**Authors:** Yoshiki Nakashima, Shinsuke Yoshida, Masayoshi Tsukahara

**Affiliations:** Kyoto University Center for iPS Cell Research and Application Foundation (CiRA Foundation), Facility for iPS Cell Therapy (FiT), Kyoto 606-8397, Japan; Kyoto University Center for iPS Cell Research and Application Foundation (CiRA Foundation), Facility for iPS Cell Therapy (FiT), Kyoto 606-8397, Japan; Kyoto University Center for iPS Cell Research and Application Foundation (CiRA Foundation), Facility for iPS Cell Therapy (FiT), Kyoto 606-8397, Japan

**Keywords:** human induced pluripotent stem cells (hiPSCs), regenerative medicine, coating material, cardiomyocyte, therapeutic cells

## Abstract

It was previously believed that human induced pluripotent stem cells (hiPSCs) did not show adhesion to the coating material Laminin 221, which is known to have specific affinity for cardiomyocytes. In this study, we report that human mononuclear cell-derived hiPSCs, established with Sendai virus vector, form peninsular-like colonies rather than embryonic stem cell-like colonies; these peninsular-like colonies can be passaged more than 10 times after establishment. Additionally, initialization-deficient cells with residual Sendai virus vector adhered to the coating material Laminin 511 but not to Laminin 221. Therefore, the expression of undifferentiated markers tended to be higher in hiPSCs established on Laminin 221 than on Laminin 511. On Laminin 221, hiPSCs15M66 showed a semi-floating colony morphology. The expression of various markers of cell polarity was significantly lower in hiPSCs cultured on Laminin 221 than in hiPSCs cultured on Laminin 511. Furthermore, 201B7 and 15M66 hiPSCs showed 3D cardiomyocyte differentiation on Laminin 221. Thus, the coating material Laminin 221 provides semi-floating culture conditions for the establishment, culture and induced differentiation of hiPSCs.

## Graphical Abstract

**Figure:**
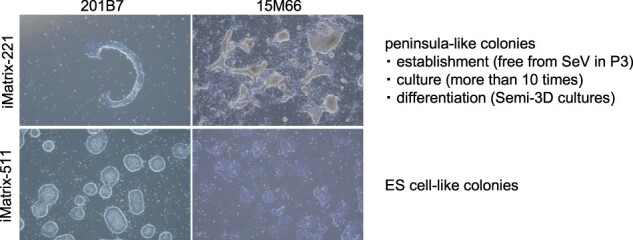

## Introduction

### Establishment of human induced pluripotent stem cells using the coating materials iMatrix-511, 411, 221 and vitronectin

Therapeutic cell products for use in regenerative medicine can be manufactured using clinical human induced pluripotent stem cells (hiPSCs) [1, 2] as the raw material; this manufacture can be divided into two main processes under Good Manufacturing Practice control [3]. The first includes methods for stocking a master cell bank of clinical hiPSCs by blood collection from patients, the establishment of hiPSCs, the selection of hiPSC clones and expansion cultures. The second involves the culture of a master cell bank of clinical hiPSCs, followed by the induction of cell differentiation to produce the desired therapeutic cells [4–6]. As the master cell bank of clinical hiPSCs is derived from blood cells from Human Leukocyte Antigen (HLA) donors homozygous for HLA-A, HLA-B and HLA-DR, it is a cell source that currently allows ∼40% of Japanese people to receive cell therapy without immune rejection [7, 8].

The clinical hiPSC master cell bank derived from HLA homozygotes uses Laminin 511 as the coating material [9]. However, Laminin 511 is not always the most suitable coating material; the selection of the coating material for use during differentiation induction and production of therapeutic cells is affected by the identity of the cell type ultimately required [10]. In addition, a single manufacturing process still needs to be developed to enable the establishment of patient-derived hiPSCs and the induction of differentiation of therapeutic cells to meet the latest medical needs for personalized medicine.

The main bottleneck, in terms of time and cost, in clinical iPSC production is the use of residual Sendai virus (SeV) vector for iPSC establishment, which requires five cell passages after initialization when Laminin 511 is used as the coating material. To overcome this difficulty, improvements in SeV vectors for iPSC establishment have been developed, such as temperature-sensitive SeV vectors [11] and auto-removal of SeV vectors [12]. The superior utility of these newly developed SeV vectors has been confirmed. However, in recent years, clinical iPSC production has been shifting from the colony-picking method, which allows production workers to arbitrarily select and sort colonies after establishment of iPSCs, to a bulk method that does not involve colony picking. The latter approach allows automation and reduces time and production costs. The bulk method requires that initialized cells are uniform iPSCs; even if the latest iPSC-establishing SeV vectors are used in actual operations, there may be overlapping factors, such as time lag in cell initialization and depth of cell initialization. The bulk method therefore cannot generate iPSCs that meet clinical iPSC criteria with fewer than five cell passages after initialization.

We performed a microscopical analysis of cultures after a number of cell initializations and found that the bulk method tends to leave behind a larger number of insufficiently initialized cells compared to the colony-picking method. Thus, although Laminin 511 is a very good coating material for clinical iPSC production by the colony-picking method, there is room for improvement in selection of the coating for clinical iPSC production with the bulk method. In particular, it will be necessary to identify and select a coating that is less prone to generation of residual, poorly initialized cells than Laminin 511 in order to advance industrial clinical iPSC production. For these reasons, the present study examined other coating materials and then focused on Laminin 221, a coating material that is capable of cell initialization and that has weak cell adhesion.

In this study, we compared hiPSC establishment, culture and differentiation induction using laminins other than Laminin 511. We selected materials that met the criteria for Japanese Standards for Biological Ingredients in order to conduct our study in a manner appropriate for clinical application. In addition to Laminin 511, the recombinant protein fragment products, Laminin 221 and Laminin 411 were selected as test materials. We also included vitronectin, a recombinant protein that is used worldwide, as a test material.

The SeV vector used for the establishment of hiPSCs does not proliferate inside the cells and is diluted by cell division; therefore, the concentration of the vector is adjusted as the multiplicity of infection (MOI) [13] so that it is lost within five passages [14]. In addition, recently developed products have been designed to automatically inactivate SeV vectors upon sensing the establishment of iPSCs [15]. Therefore, the SeV vector is at its highest concentration at the P0 stage of hiPSC establishment; the concentration decreases from P0 to P4 and is below detection sensitivity at P5.

In the second passage (P2) after establishment of hiPSCs, three types of hiPSCs are considered to coexist: (i) post-mature hiPSCs, which are mature hiPSCs that have acquired pluripotency and the ability to express endogenous initialization genes transcribed and translated from deoxyribonucleic acid (DNA); (ii) mid-maturity hiPSCs, which are hiPSCs still in the process of being established by expressing exogenous initialization genes transcribed and translated from SeV vector introduced into the cytoplasm; and (iii) early-maturity hiPSCs, which are hiPSCs whose establishment is incomplete, and cell proliferation only is activated by the expression of an exogenous initialization gene transcribed and translated from a SeV vector introduced into the cytoplasm. Green Fluorescent Protein (GFP)-negative cells (SeV vector non-remnant cells) that form embryonic stem (ES) cell-like colonies are (i) post-mature hiPSCs, GFP-positive cells (SeV vector remnant cells) that form ES cell-like colonies are (ii) mid-maturity hiPSCs, and GFP-positive cells (SeV vector remnant cells) that do not form ES cell-like colonies are (iii) early-maturity hiPSCs.

Laminin have been reported to have five types of α-chain, three types of β-chain and three types of γ-chain. These residues have the following gene names: (alpha chains) Laminin Subunit Alpha 1 (LAMA1), LAMA2, LAMA3, LAMA4, LAMA5; (beta chains) Laminin Subunit Beta 1 (LAMB1), LAMB2, LAMB3, LAMB4; (gamma chains) Laminin Subunit Gamma 1 (LAMC1), LAMC2, LAMC3 [16]. Laminin 511 is formed from the subunits α5β1γ1; Laminin 411 is formed from the subunits α4β1γ1; Laminin 221 is formed from the subunits α2β2γ1.

Laminin 511 and Laminin 411 have β1 in common: β1 has the amino acid sequence of LAMB1 (https://www.ncbi.nlm.nih.gov/protein/NP_002282.2). Laminin 511, laminin 411 and Laminin 221 have γ1 in common: γ1 has the sequence of LAMC1 (https://www.ncbi.nlm.nih.gov/protein/NP_002284.3). Laminin 511 contains α5 that has the amino acid sequence of LAMA5 (https://www.ncbi.nlm.nih.gov/protein/NP_005551.3). Laminin 411 contains α4 that has the amino acid sequence of LAMA4 (https://www.ncbi.nlm.nih.gov/protein/NP_001098676.2). Laminin 221 contains α2 that has the amino acid sequence of LAMA2 (https://www.ncbi.nlm.nih.gov/protein/NP_000417.3). Laminin 221 contains β2 that has the amino acid sequence of LAMB2 (https://www.ncbi.nlm.nih.gov/protein/NP_002283.3).

iMatrix-511, iMatrix-411 and iMatrix-221 used in this study are highly purified products of the integrin-binding site (E8 fragment) of the human laminin protein [17]. The integrin-binding site (E8 fragment) is mainly composed of α-chains. Sequencing data from PubMed (https://pubmed.ncbi.nlm.nih.gov) show that the amino acid sequences of protein subunit α5 (LAMA5) of Laminin 511, 3124–3267, 3340–3497 and 3521–3671 are identical to the Laminin G domain (LamG), an integrin β1-binding domain. Similarly, the amino acid sequences of protein subunit α4 (LAMA4) of Laminin 411, 835–1012, 1049–1207, 1237–1376, 1492–1624 and 1648–1799 are identical to the LamG, an integrin β1-binding domain. The amino acid sequences of subunit α2 (LAMA2), subunit β2 (LAMB2) and subunit γ1 (LAMC1) of Laminin 221 do not contain an integrin β1-binding domain similar to the LamG integrin β1-binding region.

Previous reports have indicated that the integrin-binding sites (E8 fragment) of Laminin 211 and Laminin 221 have an identical integrin α7β1-binding region [18]. The Laminin α2 subunit binds to dystroglycan and integrin α7β1, glycoproteins expressed on myocytes, via the laminin globular domain [19]. The molecular structures of Laminin 511, Laminin 411 and Laminin 221 differ in that Laminin 511 and Laminin 411 have an integrin β1-binding domain identified as a LamG, whereas Laminin 221 lacks LamG. Cardiac tissue has Laminin 211, Laminin 221, Laminin 411, Laminin 421, Laminin 511 and Laminin 521. Laminin 211 is the most abundantly expressed basement membrane laminin in the heart and is currently the most promising material for cardiomyocyte regeneration [20, 21]. Cardiac myocytes face the basement membrane, and the position of the basement membrane Laminin 211 in contact with cardiac myocytes is considered anatomically and histologically suitable for the structure of the culture [22].

In this study, we investigated whether Laminin 221 is a suitable coating material for culture of iPSCs for use in regenerative medicine, i.e. supports the cells in culture from establishment of iPSCs to induction of cardiomyocyte differentiation.

## Materials and methods

### Reagents

StemFit AK03N was obtained from AJINOMOTO HEALTHY SUPPLY CO., INC. (Tokyo, Japan). iMatrix-511 and iMatrix-221 were obtained from Matrixome Inc. (Osaka, Japan). A 10-mmol/l Y-27632 Solution and D-PBS (–) were obtained from Nacalai Tesque (Kyoto, Japan). TrypL™ Select Enzyme (1×), Troponin T, Cardiac Isoform Ab-1 (Clone 13-11), PSC Cardiomyocyte Differentiation Kit, and Vitronectin (VTN-N) Recombinant Human Protein, Truncated were obtained from Thermo Fisher Scientific K.K. (Kanagawa, Japan). Anti-β-actin (C4) was obtained from Santa Cruz Biotechnology (Santa Cruz, CA, USA). Anti-mouse IgG, HRP-linked Antibody was obtained from Cell Signaling Technology (Boston, MA, USA); 4% Paraformaldehyde in phosphate buffered saline (PBS) and granulocyte colony-stimulating factor (G-CSF), human, recombinant, animal-derived-free were obtained from FUJIFILM Wako Pure Chemical Corporation (Tokyo, Japan). Permeabilization Buffer (10×) was obtained from eBioscience (Vienna, Austria). CytoTune^®^-iPS 2.0 was obtained from ID Pharma Co., Ltd., (Tokyo, Japan). SRV™ iPSC-2 Vector was obtained from TOKIWA-Bio Inc. Anti-TRA-1-60, Mouse-Mono (TRA-1-60), NL557, GloLIVE, Recombinant Human Stem Cell Factor (SCF) Protein, Human, Recombinant Human Thrombopoietin (TPO) (NS0-expressed) Protein, Recombinant, Recombinant Human IL-3 Protein and Recombinant Human IL-6 Protein were obtained from R&D Systems, Inc. (Minneapolis, MN, USA). Recombinant Human Flt3-Ligand was obtained from PeproTech, Inc., Cranbury, NJ, USA. Cellstain^®^-Hoechst 33342 solution was obtained from DOJINDO LABORATORIES (Kumamoto, Japan). Foetal bovine serum (FBS) was obtained from Biosera (Kansas City, MO, USA). CytoSoft^®^ six-well Plate, Elastic Modulus 0.2, 0.5, 2, 8, 16, 32 and 64 kPa were obtained from Advanced BioMatrix, Inc. (Carlsbad, CA, USA). Other materials used were of the highest commercial grade.

### Maintenance culture of hiPSCs

The hiPSC lines 201B7 and 15M66 were established by Shinya Yamanaka (CiRA Foundation) and obtained from the CiRA Foundation (Kyoto, Japan). To culture iPSCs, a publicly available method (CiRA_Ff-iPSC_protocol_Eng_v140310) was used (https://www.cira.kyoto-u.ac.jp/j/research/img/protocol/Ff-iPSC-culture_protocol_E_v140311.pdf). Coating with both iMatrix-511 (175 μg/0.35 ml/tube) and iMatrix-221 (175 μg/0.35 ml/tube) was performed by adding 9.6 μl of each to a single well of a six-well plate. A total of 9.6 µl of both iMatrix-511 and iMatrix-221 was diluted in 1.5 ml of PBS and added as a coating solution to one well of a six-well plate. The coating time was at least 1 h at 37°C.

### Maintenance culture of human mononuclear cells

Normal Human PBMC-Japanese donor, Purified-Characterized (Lot 2010114001) (10 M cells/vial) and normal Human PBMC-Caucasian donor, Purified-Characterized (Lot: EA2177, EA2178, and EA2176) (10 million cells/vial) were obtained from FUJIFILM Wako Pure Chemical Corporation. To prepare the medium for human mononuclear cells, SCF/c-Kit ligand (final concentration 50 ng/ml), TPO (final concentration 10 ng/ml), Flt3L (final concentration 20 ng/ml), IL-6 (final concentration 50 ng/ml), IL-3 (20 ng/ml) and G-CSF (10 ng/ml) were added to a mixture of liquid A and liquid B of StemFit AK03N (AJINOMOTO HEALTHY SUPPLY CO., INC., Tokyo, Japan).

The following protocol is a method for culturing human PBMCs and is a brief description of the procedure normally followed at our institution. First, thaw a frozen vial of Normal Human PBMCs in a 37°C water bath for 1 min. Suspend the PBMCs in 5 ml of a mixture of liquid A and liquid B of StemFit AK03N, and then centrifuge the sample (440 × *g*, for 5 min at 22°C). After removing the supernatant, add 1 ml of culture medium for human mononuclear cells, mix and count the cells. A total of 3 × 10^6^ cells/ml of culture medium for human mononuclear cells was added in our case. Seed the cells in 24-well plates at a volume of 1 ml/well. Incubate at 37°C for 5 days at 5% CO_2_.

### hiPSC establishment protocol

hiPSCs were established using TOKIWA-Bio SRV iPS-2 Vector, according to the manufacturer’s instructions (TOKIWA-Bio Inc.). In brief, dispense 1 × 10^5^ cells into microtubes and centrifuge (300 × *g*, 5 min). After removing the supernatant, add 10 μl of the Vector supplied in the kit. Add 10 μl of human mononuclear cell culture medium and incubate at 37°C for 2 h. Repeat centrifugation (300 × *g*, 5 min) and wash three times using human mononuclear cell culture medium. Start the culture with human mononuclear cell medium. Add two-thirds volume of StemFit AK03N medium on Days 1, 3, 5 and 7 after the start of culture. Replace the medium with StemFit AK03N medium on Days 9, 11 and 13 after culture initiation. Perform cell passaging and colony picking from Day 15 of culture.

hiPSCs were established using CytoTune^®^-iPS 2.0 Vector, according to the manufacturer’s instructions (ID Pharma Co., Ltd., Tokyo, Japan). In brief, dispense 1 × 10^5^ cells into microtubes and centrifuge (300 × *g*, 5 min). In accordance with the data sheet attached to the kit, add 7.14 μl of Tube KOS (orange cap), 6.66 μl of Tube KLF4 (red cap) and 10.00 μl of Tube C-MYC (white cap), which is the Vector included with the kit, to 2 ml of medium for human mononuclear cells. Seed 1 × 10^5^ cells in a six-well plate. Start the culture in human mononuclear cell medium supplemented with various Vectors [MOI = 5]. After culture initiation, add two-thirds volume of StemFit AK03N medium at Days 1, 3, 5 and 7. After culture initiation, replace the medium with StemFit AK03N medium on Days 9, 11 and 13. Perform cell passaging and colony picking from Day 15 of culture.

### Cell proliferation assays

The hiPSC line 201B7 was seeded into six-well plates coated with iMatrix-511, iMatrix-411, iMatrix-221 or vitronectin at 1.3 × 10^4^ cells/well of StemFit AK03N medium, with appropriate experimental compounds (10 μM Y-27632) added. The StemFit AK03N medium was changed on Days 1, 3 and 5. The numbers of viable cells per millilitre were counted after 7 days incubation using a Countess 3FL (Thermo Fisher Scientific K.K., Kanagawa, Japan).

### Cell differentiation assays

To induce differentiation of myocardial cells, hiPSCs were cultured in six-well plates in StemFit AK03N medium to confluence on a support using a PSC Cardiomyocyte Differentiation Kit, according to the manufacturer’s instructions (Thermo Fisher Scientific K.K.). HCMs (PromoCell, Heidelberg, Germany) were used as a control.

### Real-time PCR

RNA was prepared using a SuperPREP II Cell Lysis & RT Kit for quantitative PCR (TOYOBO CO., LTD., Osaka, Japan) according to the manufacturer’s instructions. Real-time PCR was performed using a StepOnePlus system (Life Technologies, Carlsbad, CA, USA). Luna Universal qPCR Master Mix (New England Biolabs Inc., Ipswich, MA, USA) was used according to the manufacturer’s instructions. To design primers for human OCT3/4, human NANOG, human SOX2, human β-actin, human integrin α1, human integrin α5, human integrin α6, human integrin αV, human integrin β1, human integrin β3, human integrin β4, human cadherin 1 (CDH1) and human GAPDH, the gene sequences were retrieved from the US National Library of Medicine National Institutes of Health (NIH) website (https://www.ncbi.nlm.nih.gov/pubmed/). The primers for human OCT3/4, human NANOG, human SOX2, human β-actin, human integrin α1, human integrin α5, human integrin α6, human integrin αV, human integrin β1, human integrin β3, human integrin β4 and human CDH1 and human GAPDH were designed using the Primer 3 Plus application (http://www.bioinformatics.nl/cgi-bin/primer3plus/primer3plus.cgi). The primer sequence for HERV-K Gag CA was selected from the sequence described in a previous paper [26]. Other primers were purchased from Takara Bio Inc. (Shiga, Japan).

The primers used for PCR were as follows:

human OCT3/4 (NM_002701.4) 144 bp

(forward) GACAGGGGGAGGGGAGGAGCTAGG

(reverse) CTTCCCTCCAACCAGTTGCCCCAAAC

human NANOG (NM_024865.2) 391 bp

(forward) CAGCCCCGATTCTTCCACCAGTCCC

(reverse) CGGAAGATTCCCAGTCGGGTTCACC

human SOX2 (NM_003106.2) 151 bp

(forward) GGGAAATGGGAGGGGTGCAAAAGAGG

(reverse) TTGCGTGAGTGTGGATGGGATTGGTG

human β-actin (NM_001101.3) 224 bp

(forward) GTGACATTAAGGAGAAGCTGTGCTA

(reverse) CTTCATGATGGAGTTGAAGGTAGTT

HA115387(SCRIB)

(forward) TTCCAGACCTGTCTATGACATCCA

(reverse) CCAGAAGCACCAGAGCCACT

HA324529(TJP1)

(forward) GCACGGGCATTGTTTAATGTC

(reverse) GGATTCAGTCCACAAAGGTGTTTAC

HA327917(CRB3)

(forward) AGACCACTTCTGCAAATGAGAATAG

(reverse) GAAGACCACGATGATAGCAGTGA

HA370266(PARD3)

(forward) ATCTCGGTGGCTCCCATCTTC

(reverse) TTTCCACATCGGGATTCACAG

HA366188(CTNNA1)

(forward) GATGTCATCAGTGCTGCCAAGA

(reverse) GCTGCAGATGTTCAGCTGGTG

HA162584(CTNNB1)

(forward) CATCCTAGCTCGGGATGTTCAC

(reverse) TCCTTGTCCTGAGCAAGTTCAC

HA391542(OCLN)

(forward) TGCTTCAGCATCAGGATGGA

(reverse) TGGTGTAGTGTAGCAGAATCATGAG

human integrin α1(NM_181501.1) 234 bp

(forward) CAATGAGACAGTCCCTGAAGTTATT

(reverse) GAGTTGATACTGAAAGGATCCTCAA

human integrin α5(NM_002205.4) 229 bp

(forward) CTGCTACCTCTCCACAGATAACTTC

(reverse) GATCAGGTACTCGGGGTAATAAGAT

human integrin α6(NM_000210.3) 195 bp

(forward) GTTTTGTTTCCTCCCCTATCTGTAT

(reverse) GCTCCCCATATAACTTAACATTGTG

human integrin αV(NM_001144999.2) 191 bp

(forward) AGATTAGACAGAGGAAAGAGTGCAA

(reverse) ACATTAGTGGTAACCAATGTGGAGT

human integrin β1(NM_002211.3) 179 bp

(forward) CTGAAGACTATCCCATTGACCTCTA

(reverse) GCTAATGTAAGGCATCACAGTCTTT

human integrin β3(NM_000212.2) 156 bp

(forward) GCACTTAATGATAAGCAGTCATCCT

(reverse) CACACTTCCACATACTGACATTCTC

human integrin β4(NM_000213.4)220 bp

(forward) CTTTGCTGTCACCAACTACTCCTAT

(reverse) AGTCCTCGTCTTCTGGAACATCT

HA381019(NECTIN1)

(forward) GGAATGGTCTGATCCTGCTGAA

(reverse) ACCAGTGACTTGGGCAAGTG

HA169657(NECTIN2)

(forward) GAGCACAGCCCACTCAAGAC

(reverse) AAGGTGGGCAGCTCATGGTA

HA248967(NECTIN3)

(forward) GGTGACCTGGAMGCCACAAGT

(reverse) ACAGTGCAATGCTTCCCAAAGTC

human CDH1 (NM_004360.4) 191 bp

(forward) GCCACATCTTGACTAGGTATTGTCT

(reverse) GCAGCACTTTAGGCACTATTCTAAG

human GAPDH (NM_001256799.2) 175 bp

(forward) AAGAGCACAAGAGGAAGAGAGAGAC

(reverse) GTTGAGCACAGGGTACTTTATTGAT

HERV-K Gag CA

(forward) CAAGACCCAGGAAGTACCT

(reverse) ACACTCAGGATTGGCGTT

### Western blotting

Western blotting analyses using ATTO Products (EzRIPA Lysis kit, cPAGE Twin, myPower II 300, HorizeBLOT 2M-R, EzApply, EzStandard PrestainBlue, c-PAGEL 10%, EzRun, P plus membranes, Filter paper, EzFastBlot, EzBlock Chemi, EzTBS and EzWestBlue) were performed according to the manufacturer’s instructions (ATTO, Tokyo, Japan). Blots were probed using specific antibodies for Troponin T and β-actin. Images were quantified using the NIH Image J software program (Version 1.53; https://imagej.nih.gov/ij).

### Immunofluorescence staining

Cells were washed with PBS and then immersed in 4% paraformaldehyde PBS and fixed for 10 min. Permeabilization Buffer (10×) from eBioscience (Vienna, Austria) was used instead of 0.1% Triton X-100 in PBS. Liquid 1% FBS added to PBS was used as an antibody diluent.

Immunofluorescence staining was performed using specific antibodies for Troponin T, Cardiac Isoform Ab-1 (Clone 13-11; Thermo Fisher Scientific K.K.), Anti-β-actin (C4; Santa Cruz Biotechnology) and Anti-mouse IgG, HRP-linked Antibody (Cell Signaling Technology). Cellstain^®^-Hoechst 33342 solution (DOJINDO LABORATORIES, Kumamoto, Japan) was used for nuclear staining of the cells. Images were recorded using a fluorescence microscope (BZ-X800; Keyence, Osaka, Japan). Since SeV is carried on the SeV vector, SeV-positive cells can be positively identified with a GFP light source without the need for fluorescently labelled antibodies. Tra-1-60 was then purchased from R&D Systems, Inc. and used for live-staining. After fluorescent staining, cells were detached with trypsin according to the usual cell detachment method and then counted after making cell suspensions with StemFit AK03 containing ROCK inhibitor (10 μM).

### Statistical analyses

Statistical analyses were performed using Student’s *t*-test to compare two samples. Statistical analyses comparing more than two groups were performed using a one-way analysis of variance with the StatPlus software program (AnalystSoft Inc., Walnut, CA, USA). Statistical significance was set at *P *<* *0.05 for all tests.

## Results

We determined whether iPSCs could be established using four different coating materials (iMatrix-511, iMatrix-411, iMatrix-221 and vitronectin) and compared differences in colony morphologies and messenger ribonucleic acid (mRNA) expression patterns.

Japanese donor-derived mononuclear cells were exposed to SeV vectors (SRV™ iPSC-2 Vector, TOKIWA-Bio Inc., Ibaraki, Japan) carrying four human-derived initialization genes [2, 23] (octamer-binding transcription factor [OCT3/4], Kruppel-like factor 4 [KLF4], sex determining region Y-box 2 [SOX2] and c-MYC) were exposed for 2 h at 37°C to introduce the vectors into the cytoplasm. Vector-transfected cells were then seeded on iMatrix-511, iMatrix-411, iMatrix-221 and vitronectin coating materials.

**Figure 1.**
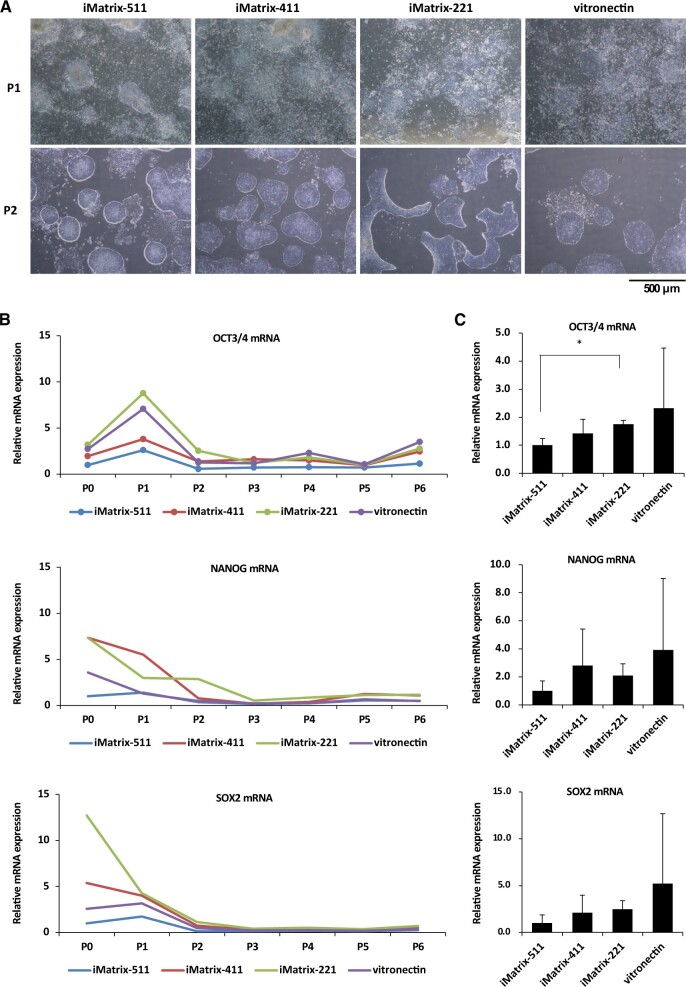
Establishment of hiPSCs using Sendai virus vectors (SRV™ iPSC-2 Vector) and various coating materials. (**A**) The morphology of hiPSCs established in the presence of StemFit AK03N at passage number 1 (upper panels) and 2 (lower panels) in the presence of iMatrix-511, iMatrix-411, iMatrix-221 and vitronectin. Optical microscope images are shown. Scale bar = 500 μm. (**B**) The time course of mRNA expression of OCT3/4 (top panel), NANOG (Middle panel) and SOX2 (lower panel) in colonies at passage numbers 0–6 after iPSCs were established. The *y*-axis represents the relative position of the mRNA expression of the target gene expressed at P0 by hiPSCs established on iMatrix-511 as 1 (*n* = 1). (**C**) *OCT3/4* (top panel), *NANOG* (middle panel) and *SOX2* (lower panel) mRNA expression in hiPSCs on iMatrix511, iMatrix-411, iMatrix-221 and vitronectin coating was measured by real-time PCR. Values indicate the relative value obtained by converting the calculated value of iMatrix-511–1 (*n* = 3). The data are presented as the mean ± standard error. *P < 0.05.

Colonies of established hiPSCs were observed after 14 days. All clones were passaged in bulk, without picking colonies, to establish the passage 1 (P1) culture. A stealthy ribonucleic acid (RNA) vector was designed to be automatically erased in response to the expression of microRNA-302 [12, 24, 25] in the hiPSCs. Optical micrographs of P1 cultures showed polygonal colonies and proliferation of fibroblast-like cells around them in cultures using the iMatrix-511, iMatrix-411, iMatrix-221 and vitronectin coating (Fig. 1A, upper panels**)**. At P2, ES cell-like colonies were present in cultures using iMatrix-511, iMatrix-411 and vitronectin coating; in contrast, peninsular-like colonies were observed in the iMatrix-221 coating (Fig. 1A, lower panels). In subsequent passages, when the density of cells to be seeded was high, hiPSC colonies cultured on iMatrix-511, iMatrix-411, iMatrix-221 and vitronectin coating showed similar morphologies with adhesion between colonies. However, when the density of cells to be seeded was low, the hiPSC colonies cultured on iMatrix-511, iMatrix-411 and vitronectin had an ES cell-like morphology, whereas those on iMatrix-221 tended to show a peninsular-like morphology. Expression of undifferentiated markers (OCT3/4, NANOG, SOX2) was analysed by sampling cells at each passage from P0 to P6 in bulk passaged hiPSCs. Higher levels of expression of the undifferentiated markers was observed in bulk hiPSCs cultured with iMatrix-221, iMatrix-411 and vitronectin from P0 to P2 than in bulk hiPSCs cultured with iMatrix-511 (Fig. 1B, upper, middle and lower panels).

To analyse this difference in gene expression in more detail, we prepared three samples from bulk cells sampled for passaging in the bulk of P0 and analysed expression of the markers OCT3/4, NANOG and SOX2 using a real-time polymerase chain reaction (PCR). The results showed that *OCT3/4* mRNA expression was significantly higher in bulk hiPSCs cultured with iMatrix-221 than in bulk hiPSCs cultured with iMatrix-511 (Fig. 1C, upper panel). Next, iPSC establishment was performed using peripheral blood mononuclear cells (PBMCs) from three different Caucasian subjects in triplicate using iMatrix-511-, iMatrix-411-, iMatrix-211- and vitronectin-coated wells to generate P0, P1, P2 and P3 samples. The expression of the markers OCT3/4, NANOG and SOX2 was analysed by sampling cells at each passaging step from P0 to P3 in bulk-passaged hiPSCs. iPSCs established in iMatrix-221-coated wells were found to have a significantly higher level of expression of OCT3/4 than iPSCs established on wells coated with other materials at P1. Furthermore, iPSCs established on iMatrix-411-coated wells had a significantly higher level of expression of OCT3/4 than iPSCs established on wells coated with other materials at P3. In contrast, iPSCs established on vitronectin-coated wells had a significantly lower level of expression of OCT3/4 than iPSCs established on wells coated with iMatrix-511 and iMatrix-221 at P2 (Supplementary Fig. S1A). The expression of NANOG was significantly higher in iPSCs established on iMatrix-411-coated wells compared to those on other materials at P3 (Supplementary Fig. S1B). In addition, iPSCs established on iMatrix-221-coated wells had significantly higher expression of SOX2 at P1 than iPSCs established on wells coated with other materials. Peak expression of SOX2 was observed at P2 for iPSCs established on iMatrix-511-, iMatrix-411- and vitronectin-coated wells, but at P1 for iPSCs on iMatrix-221-coated wells (Supplementary Fig. S1C).

Next, we examined residual SeV vectors levels in cells using MOP104,105 primers. Residual levels of SeV remained constant in iPSCs established on iMatrix-511-, iMatrix-411- and vitronectin-coated wells from P0 to P3. In contrast, iPSCs established on iMatrix-221-coated wells showed a peak level of SeV at P1 and a subsequent decrease from P2. Thus, iPSCs established on iMatrix-221-coated wells had lower SeV residual levels at P2 and P3 than those established on wells coated with other materials (Supplementary Fig. S1D). Recently, it was reported that SOX2, an important marker of initialization and pluripotency in undifferentiated cells, induces genome expression in human endogenous retroviruses (HERVs) [26]. We investigated the initialization state of iPSCs by analysing expression of HERVs. Theoretically, HERV genome expression should increase when expression of SOX2 is at its highest level. We used the HERV-K Gag CA described in the literature as the primer for the viral genome of HERVs. The analysis showed that peak HERV-K Gag CA expression in iPSCs established on iMatrix-511- and iMatrix-221-coated wells had coincided with the passages at which *SOX2* mRNA expression peaked. Expression of HERV-K Gag CA at P2 was significantly higher in iPSCs established on iMatrix-221-coated wells than in wells coated with iMatrix-411 and vitronectin. In contrast, peak *SOX2* mRNA expression in iPSCs established on iMatrix-511-coated wells and iMatrix-221-coated wells did not coincide with peak HERV-K Gag CA expression, and no strong peaks were observed (Supplementary Fig. S1E). The viral genome of HERVs accounts for 8% of total human DNA and has been suggested to have a protective effect on DNA. Further studies on the relationship between HERV genome expression and DNA mutations involved in iPSC initiation is warranted.

### The evaluation of hiPSC established with coating materials iMatrix-511, 411, 221 and vitronectin

**Figure 2.**
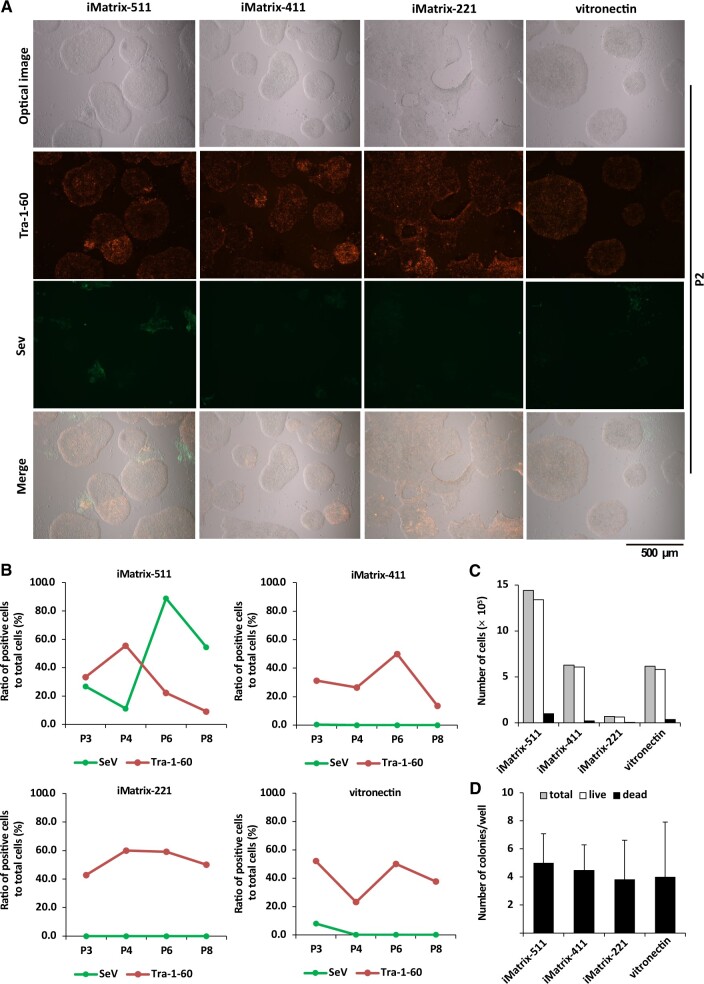
Evaluation of hiPSCs established using Sendai virus vectors (SRV™ iPSC-2 Vector) and various coating materials. (**A**) hiPSCs established in the presence of StemFit AK03N at passage number 2 in the presence of iMatrix-511, iMatrix-411, iMatrix-221 and vitronectin. Optical microscope images are shown (top panels). The cells were immunostained using Tra-1-60 antibodies (second from the top) and GFP to detect Sendai virus vectors remaining in the cells (third from the top). The bottom panels show merged images. Scale bar = 500 μm. (**B**) Data from live staining of cells are shown. The time course of the proportion of cells (%) expressing Sendai virus vectors remaining in the cells and Tra-1-60 positive cells in colonies at passage numbers 3–8 after iPSCs were established in the presence of iMatrix-511, iMatrix-411, iMatrix-221 and vitronectin (*n* = 1). (**C**) Cell proliferation assay. The total numbers of hiPSCs (grey bar), the number of live cells (white bar) and the number of dead cells (black bar) were counted at 7 days after seeding the cells of the seventh generation of passages at a cell density of 1.3 × 10^4^ cells/well (six-well plates) in the presence of StemFit AK03N on iMatrix-511, iMatrix-411, iMatrix-221 and vitronectin coating (*n* = 1). (**D**) The number of hiPSC colonies established using Sendai virus vectors (CytoTune^®^-iPS 2.0) was counted. The number of hiPSC colonies established on iMatrix-511, iMatrix-411, iMatrix-221 or vitronectin was counted at 14 days after Sendai virus infection (CytoTune^®^-iPS 2.0) (*n* = 6). The data are presented as the mean ± standard error.

Scaffold materials iMatrix-511, iMatrix-411, iMatrix-221 and hiPSCs of the P2 established on vitronectin were stained, and fluorescence immunostaining was used to identify GFP-positive cells (SeV vector remnant cells) and GloLIVE NL557-positive cells (Tra-1-60-positive cells) (Fig. 2A).

hiPSCs from P3 to P8 that had been established on iMatrix-511, iMatrix-411, iMatrix-221 and vitronectin were stained to determine the number of GFP-positive cells (SeV vector remnant cells) and GloLIVE NL557-positive cells (Tra-1-60-positive cells), and the proportions of positive cells in the total cell count were obtained (Fig. 2B). The results showed that hiPSCs established on iMatrix-511 had a greater proportion of SeV vector remnant cells from P3 to P8 compared to cells established on iMatrix-411, iMatrix-221 and vitronectin.

The fluorescence microscopy analysis showed that more GFP-positive early-maturity hiPSCs were present on iMatrix-511 at P2 than on iMatrix-411, iMatrix-221 and vitronectin (Fig. 2A, leftmost panel, third and fourth row from the top). A quantitative evaluation of the rate of early-maturity hiPSCs showed that 26.7% were present at P3, 11.1% at P4, 88.9% at P6 and 54.5% at P8 in cultures on iMatrix-511 (Fig. 2B, top left panel). In cultures on iMatrix-411, only 0.4% of positive cells were observed at P3 (Fig. 2A, top right panel); on vitronectin, 8% positive cells were found at P3 (Fig. 2A, lower right panel). On iMatrix-221, no positive cells were observed at cell passages P3 to P8 (Fig. 2A, lower left panel). Clinical hiPSCs are rejected for use due to quality control safety concerns [27] if they are contaminated with early-maturity hiPSCs.

Previous studies have reported no significant differences in morphology, in expression of pluripotency markers or in gene expression profiles in hiPSCs established by bulk passaging of clones compared to those established with clones selected by colony picking [28]. Expression of the pluripotent stem cell marker Tra-1-60 [29] identifies post-mature hiPSCs. According to a report comparing international quality assessment criteria for clinical hiPSCs [30], the quality threshold for human ES cells is a Tra-1-60 positivity rate of 50% [31, 32], while that of hiPSCs is a Tra-1-60 positivity rate of ≥70% [33, 34], although this may vary depending on the guidelines of each country. Here, we found a Tra-1-60 positivity rate for hiPSCs established on iMatrix-221 of 42.9% at P3, 60.0% at P4, 59.1% at P6 and 50.0% at P8 (Fig. 2B, lower left panel). The expression of Tra-1-60 was confirmed by fluorescence immunostaining of P2 hiPSCs when the initialization gene expression vector was present in the cytoplasm. Interestingly, peninsular colonies in cultures on iMatrix-221 had a layer of cells with high Tra-1-60 expression in the morphologically curved region. hiPSCs established on vitronectin showed ES cell-like colonies with uniform Tra-1-60 expression at P2. In contrast, P2 hiPSCs on iMatrix-511 and iMatrix-411 showed ES cell-like colonies with heterogeneous Tra-1-60 expression among the colonies (Fig. 2A, second panels from the top).

The numbers of total cells, viable cells and dead cells were cell counted on Day 7 after seeding P7 cells at a density of 1.3 × 10^4^ cells/well (six-well plates). The hiPSCs proliferated more than 100-fold on iMatrix-511, whereas they proliferated more than 40-fold on iMatrix-411 and vitronectin. However, on iMatrix-221, the hiPSCs only proliferated just over 4-fold (Fig. 2C).

To confirm the efficiency of hiPSC establishment, we used another SeV vector (CytoTune^®^-iPS 2.0, ID Pharma Co., Ltd., Japan) to initialize human mononuclear cells for hiPSC establishment. On Day 14 after SeV infection, an average of four colonies had established on iMatrix-511, iMatrix-411, iMatrix-221 and vitronectin cultures (Fig. 2D).

Whether the formation of semi-floating cell populations is caused by active signalling from iMatrix-221 or is simply a function of the low adhesiveness of iMatrix-221 is unclear. In addition, it is unclear whether cell–cell interactions predominate over cell–surface interactions in these semi-floating cell populations. Furthermore, since many cell types form similar cell clusters on low-adhesion surfaces, it needs to be determined whether the same effect is elicited in uncoated wells.

iPSCs produced in bulk on Laminin 511 are contaminated with iPS-like cells and with cells that have acquired a proliferative ability that is closer to somatic cell characteristics than iPS-like cells. These cells show the ability to adhere not only to Laminin 511 but also to the bottom surface of non-coated culture vessels. In order to create a method for generating cells with a proliferative potential that more closely resembles that of somatic cells than iPS-like cells, we established iPSCs by combining StemFit AK03 (Ajinomoto Co., Inc., Tokyo, Japan) medium and an artificial synthetic coat material similar in composition to vitronectin. After the establishment of iPSCs, P4 cells were detached using only EDTA and seeded onto a non-coated dish. At 48 h after cell seeding, the culture supernatant was aspirated and non-adherent cells were collected. The non-adherent cells were seeded onto iMatrix-511-coated dishes (Supplementary Fig. S2A, above illustration). Cells that adhered to the non-coated dish (a) (Supplementary Fig. S2A, left-lower panel) and those that did not adhere to the non-coated dish but subsequently adhered to the iMatrix-511-coated dish (b) were analysed microscopically (Supplementary Fig. S2A, right-lower panel). Since iPSCs do not have the ability to adhere to non-coated dish surfaces, we expected purified iPSCs to be recovered from dish (b). However, iPSCs and iPS cell-like cells with various colony morphologies, as indicated by white arrows, were observed on dish (b) (Supplementary Fig. S2A, right-lower panel). Cells from the dish (b) were further separated into four groups (1–4) using a non-coated dish and an iMatrix-511-coated dish based on differences in cell adhesion characteristics (Supplementary Fig. S2B). Optical micrographs of the four cell groups (1–4) showed a mixture of colony-like and fibroblast-like cells without specific colony morphology characteristics in each group (Supplementary Fig. S3A). To characterize the four groups of cells, mRNA was extracted from cell pellets and PCR samples were prepared for TaqMan Array analysis (Human Stem Cell Pluripotency). The mRNA expression of the research cell line 201B7 cultured on iMatrix-511 was set at 1 to normalize the data. The expression of each factor in the ‘Expression in undifferentiated cells’ (Supplementary Fig. S3B), ‘Maintenance of pluripotency’ (Supplementary Fig. S3C) and ‘Correlation to stemness’ (Supplementary Fig. S3D) panels was examined as to evaluate the level of cell initialization. The expression of each factor in the ‘Expression in undifferentiated cells’, ‘Maintenance of pluripotency’ and ‘Correlation to stemness’ panels tended to be highest in Group 1 and lowest in Group 4, indicating that the expression of each factor in the ‘Expression in undifferentiated cells’, ‘Maintenance of pluripotency’ and ‘Correlation to stemness’ panels was the same as in Group 1. Group 4 tended to show the lowest values.

Next, we examined the expression of each factor in a ‘Differentiation markers’ panel (Supplementary Fig. S3E) as an indicator of the level of cell differentiation. The expression of each factor in this panel for cells in Groups 1–4 were similar. The results showed that mRNA expression of undifferentiated markers tended to be higher overall in cells that adhered to the non-coated dish than in cells that did not adhere to the iMatrix-511-coated dish. The difference in adhesion characteristics was not caused by the induction of cell differentiation but was partly attributable to differences in the undifferentiated state of the cells. iMatrix-511-coated dishes and non-coated dishes thus cannot be used to remove iPS-like cells or fibroblast-like cells generated in the iPSC establishment process using cell adhesion properties; iMatrix-511-coated and non-coated dishes are not suitable for the removal of iPS-like cells. Uniform iPSC colonies could not be purified in this instance.

### Colony morphology and analyses of hiPSCs cultured on coating materials iMatrix-511 and iMatrix-221

**Figure 3.**
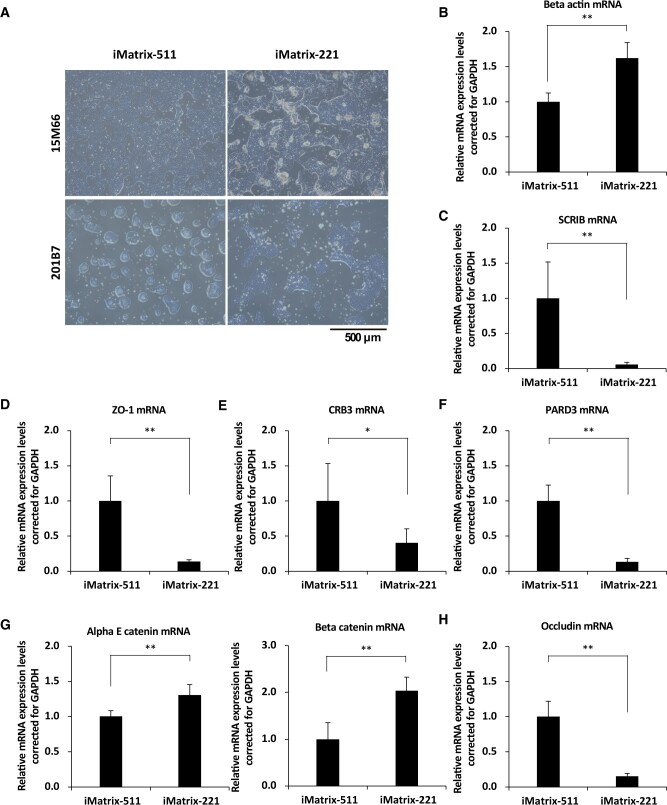
The evaluation of cell polarity. (**A**) The morphologies of hiPSC line 15M66 cells (upper panels) and 201B7 cells (lower panels) in cultures 3–4 days after seeding at 5 × 10^4^ cells/well (six-well plates) or 5–7 days after seeding at 5 × 10^4^ cells/well (six-well plates) in the presence of StemFit AK03N and iMatrix-511 (left panels) or iMatrix-221 (right panels). Optical microscope images are shown. Scale bar = 500 μm. (**B**) Expression of β-actin, (**C**) SCRIB, (**D**) ZO-1, (**E**) CRB3, (**F**) PARD3, (**G**, right) α-E catenin, (**G**, left) β-catenin and (**H**) *Occludin* mRNA in hiPSCs on iMatrix511 or iMatrix-221 was measured by real-time PCR. Values indicate the relative value obtained by converting the calculated value of iMatrix511 to 1 (*n* = 3). The data are presented as the mean ± standard error. *P < 0.05, **P < 0.01.

The morphologies of hiPSC colonies in cultures on iMatrix-511 and iMatrix-221 were investigated using optical microscopy images. Human epithelial fibroblast-derived 201B7 cells and human mononuclear cell-derived 15M66 cells (research strain of clinical hiPSCs) were obtained 3–4 days after seeding at a cell concentration of 1 × 10^5^ cells/well (six-well plates) or 5–7 days after seeding at a cell concentration of 5 × 10^4^ cells/well (six-well plates). Colony morphologies of hiPSCs were not ES cell-like for 15M66 cells cultured on iMatrix-511 and iMatrix-221, even though this colony morphology is usually observed when 15M66 cells are seeded at high cell concentrations. Here, the 15M66 cells produced a large number of semi-floating cell clumps attached to the colonies of hiPSCs when cultured on iMatrix-221 (Fig. 3A, upper panels).

Colony morphologies of hiPSCs produced in strain 201B7 cultures on iMatrix-511 and iMatrix-221 were analysed. Of note, hiPSCs cultured on iMatrix-221 showed colonies with a rhombus or peninsular-like morphology. However, hiPSCs cultured on iMatrix-511 showed colonies with ES cell-like morphology (Fig. 3A, lower panels). In 2007, Krtolica *et al*. generated a model of human ES cells with reduced cell polarity by re-applying Matrigel to the cells [35]. At that time, the expression of zona occludens-1 (ZO-1) was used as an indicator of cell polarity. The cell polarity-deprived human ES cells showed a peninsular-like colony morphology, similar to that of hiPSCs cultured on iMatrix-221 in the present study.

To investigate the cause of the difference in colony morphology between hiPSCs cultured on iMatrix-511 and iMatrix-221, we performed an mRNA expression analysis of various factors related to cell polarity. Abagnale *et al*. hypothesized in 2017 that cell polarity influences the surface shape of iPSC colonies [36]. It is known that cell polarity is strongly involved in the critical early stages of human embryonic development, and the importance of cell polarity has also been reported in 3D human pluripotent stem cell-derived cysts (hPSC-cysts) [37]. Cell polarity is the spatial polarity of a cell. It refers to the distribution of characteristic intracellular components, such as the plasma membrane and cytoskeleton, with different protein and lipid contents. The most common example of cell polarity is epithelial cell polarity; the 201B7 cell line is derived from epithelial fibroblasts.

Cell polarity has previously been evaluated in mathematical modelling studies. Cell shape control and substrate stiffness sensitivity drive the organization and local alignment of actin fibres and the cytoskeleton. Increased substrate stiffness leads to the formation of stronger actin fibres within the cell. In contrast, cell shape determines the local arrangement of actin fibres in the cell periphery [38]. According to this model, a decrease in cell polarity would lead to a reduced ability to form strong actin fibres and to adapt to a lower-stiffness substrate surface. Mathematical models also suggest that stronger intercellular adhesion makes it easier for colonies to aggregate and form a circle [39]. In the present study, the decreased expression of intercellular adhesion factors was observed in hiPSCs cultured on iMatrix-221 but not on iMatrix-511. Specifically, decreased expression was found for ZO-1 (Fig. 3D) and Occludin (Fig. 3H) at tight junctions, Poliovirus receptor-related 1 (PVRL1) (Fig. 5A), Poliovirus receptor-related 3 (PVRL3) (Fig. 5B), Nectin2 (Fig. 5C), E-cadherin (Fig. 5D) at adherens junctions, and SCRIB (Fig. 3C) in the basolateral membrane. These changes in expression suggest a decrease in cell polarity. The increased expression of intercellular adhesion factors in hiPSCs cultured on iMatrix-511 but a decrease in expression in hiPSCs cultured on iMatrix-221 provide a logical explanation for the influence of these factors on colony morphology. Our observations support the interpretation that intercellular adhesion is a factor that affects colony morphology.

Next, we investigated epithelial cell polarity [40–43], which is known to be influenced by several factors: the apical membrane, the basolateral membrane, tight junctions and adherens junctions.

#### Organization of polarity

β-actin is a marker of polarity [44, 45]. We examined *β-actin* mRNA expression in hiPSCs (201B7 cell line) cultured on iMatrix-511 and iMatrix-221. We found that *β-actin* mRNA expression was significantly increased when iMatrix-221 was used as the coating material compared with iMatrix-511 (mean [standard deviation]: iMatrix-511, 1.00 [0.12]; iMatrix-221, 1.62 [0.22]; *P* = 2.0E-04; *n* = 6) (Fig. 3B).

#### Basolateral membrane formation

Scribble planar cell polarity protein (SCRIB) is a marker of basolateral membrane formation [46, 47]. Analysis of *SCRIB* mRNA expression in hiPSCs (201B7 cell line) showed that expression was significantly decreased when iMatrix-221 was used as the coating material compared with iMatrix-511 (mean [standard deviation]: iMatrix-511, 1.00 [0.52]; iMatrix-221, 0.06 [0.03]; *P* = 2.0E-03; *n* = 6) (Fig. 3C).

#### Tight junction formation

ZO-1, also known as Tight junction protein-1 (TJP1), is a marker of tight junction formation [48, 49]. Analysis of *ZO-1* mRNA expression in hiPSCs (201B7 cell line) showed that expression was significantly decreased when iMatrix-221 was used as the coating material compared with iMatrix-511 (mean [standard deviation]: iMatrix-511, 1.00 [0.36]; iMatrix-221, 0.14 [0.02]; *P* = 7.6E-05; *n* = 6) (Fig. 3D).

#### Apical transmembrane protein

Crumbs cell polarity complex component 3 (CRB3) is a marker of apical transmembrane protein [50, 51]. Analysis of *CRB3* mRNA expression in hiPSCs (201B7 cell line) showed that expression was significantly decreased when iMatrix-221 was used as the coating material compared with iMatrix-511 (mean [standard deviation]: iMatrix-511, 1.00 [0.53]; iMatrix-221, 0.41 [0.20]; *P* = 0.03; *n* = 6) (Fig. 3E).

#### Apical membrane formation

Par-3 family cell polarity regulator (PARD3) is a marker of apical membrane formation [52, 53]. Analysis of *PARD3* mRNA expression of PARD3 in hiPSCs (201B7 cell line) showed that expression was significantly decreased when iMatrix-221 was used as the coating material compared with iMatrix-511 (mean [standard deviation]: iMatrix-511, 1.00 [0.23]; iMatrix-221, 0.13 [0.05]; *P* = 5.7E-06; *n* = 6) (Fig. 3F).

#### Linking adherens junctions with actin cytoskeleton

α-E catenin (Catenin α-1 [CTNNA1]) and β-catenin (Catenin β-1 [CTNNB1]) are markers linking adherens junctions with the actin cytoskeleton [54, 55]. Analysis of α-E catenin and *β-catenin* mRNA expression in hiPSCs (201B7 cell line) showed that expression of *α-E catenin* mRNA was significantly increased when iMatrix-221 was used as the coating material compared with iMatrix-511 (mean [standard deviation]: iMatrix-511, 1.00 [0.08]; iMatrix-221, 1.30 [0.15]; *P* = 6.1E-03; *n* = 6) (Fig. 3G, left panel); likewise, *β-catenin* mRNA expression was significantly increased when iMatrix-221 was used as the coating material compared with iMatrix-511 (mean [standard deviation]: iMatrix-511, 1.00 [0.35]; iMatrix-221, 2.03 [0.29]; *P* = 3.7E-04; *n* = 6) (Fig. 3G, right panel).

#### Tight junction protein

Sapiens occludin (OCLN) is a tight junction marker protein [56, 57]. Analysis of *OCLN* mRNA expression in hiPSCs (201B7 cell line) showed that expression was significantly decreased when iMatrix-221 was used as the coating material compared with iMatrix-511 (mean [standard deviation]: iMatrix-511, 1.00 [0.22]; iMatrix-221, 0.15 [0.04]; *P* = 4.9E-06; *n* = 6) (Fig. 3H).

We previously reported that the coating material Laminin 511 binds to α3β1, α6β1 and α6β4 integrins on the cell membrane and activates PI3K/AKT- and Ras/MAPK-dependent signalling pathways. hiPSCs are protected from apoptosis through the interaction of the Laminin 511/α6β1 integrin with the intercellular adhesion molecule E-cadherin [58].

#### Basal membrane integrity

**Figure 4.**
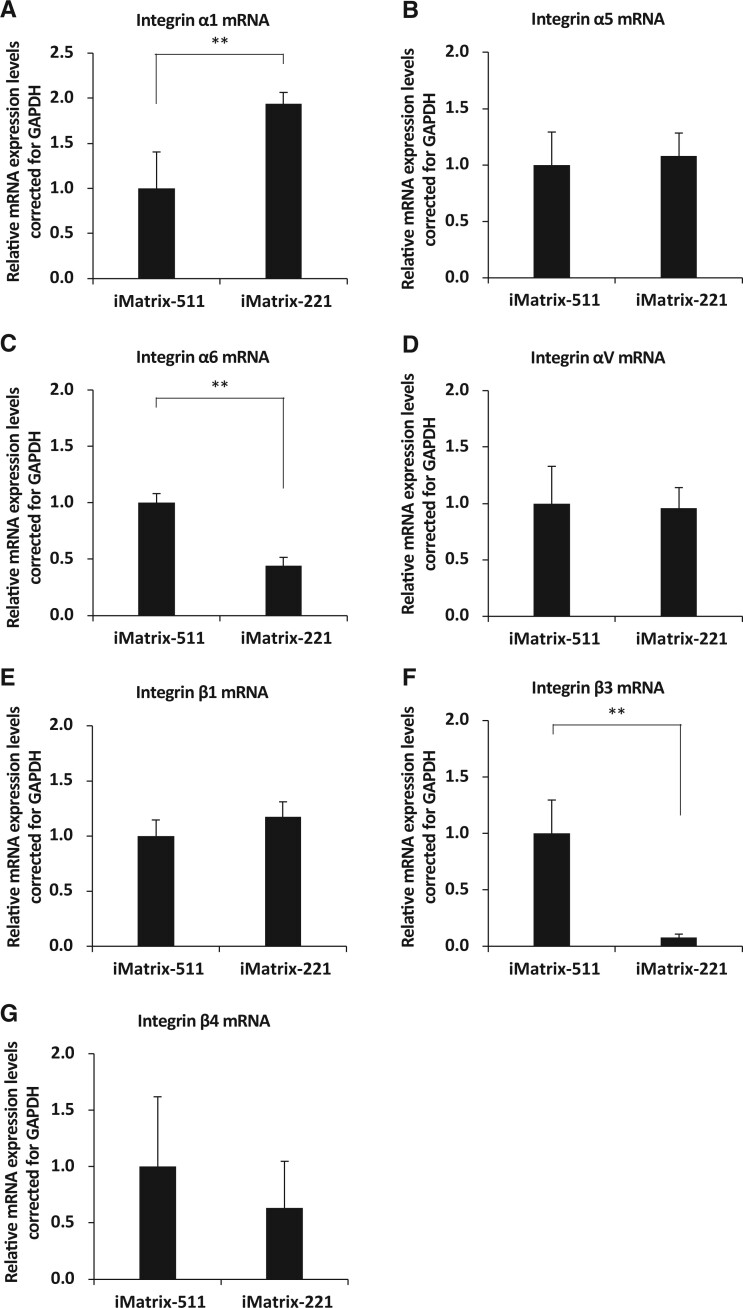
The evaluation of integrin α, an ECM receptor. (**A**) *Integrin α1*, (**B**) *Integrin α5*, (**C**) *Integrin α6*, (**D**) *Integrin αV*, (**E**) *Integrin β1*, (**F**) *Integrin β3* and (**G**) *Integrin β4* mRNA expression from hiPSCs on iMatrix511 or iMatrix-221 was measured by real-time PCR. Values indicate the relative value obtained by converting the calculated value of iMatrix511 to 1 (*n* = 3). The data are presented as the mean ± standard error. **P < 0.01.

Integrin α (Integrin α1, Integrin α5, Integrin α6 and Integrin αV) and Integrin β (Integrin β1, Integrin β3 and Integrin β4) are markers of basal membrane integrity [59, 60]. Analysis of the mRNA expression of various integrins in hiPSCs (201B7 cell line) showed that *Integrin α1* mRNA expression was significantly increased when iMatrix-221 was used as the coating material compared with iMatrix-511 (mean [standard deviation]: iMatrix-511, 1.00 [0.40]; iMatrix-221, 1.94 [0.13]; *P* = 4.2E-04; *n* = 6) (Fig. 4A). In contrast, *Integrin α5* mRNA expression was similar in cultures on both iMatrix-511 and iMatrix-221 (mean [standard deviation]: iMatrix-511, 1.00 [0.29]; iMatrix-221, 1.08 [0.21]; *P* = 0.62; *n* = 6) (Fig. 4B). *Integrin α6* mRNA expression was significantly decreased when iMatrix-221 was used as the coating material compared with iMatrix-511 (mean [standard deviation]: iMatrix-511, 1.00 [0.08]; iMatrix-221, 0.44 [0.08]; *P* = 3.7E-07; *n* = 6) (Fig. 4C). In contrast, *Integrin αV* mRNA expression was similar in cultures on both iMatrix-511 and iMatrix-221 (mean [standard deviation]: iMatrix-511, 1.00 [0.33]; iMatrix-221, 0.96 [0.18]; *P* = 0.80; *n* = 6) (Fig. 4D).

*Integrin β1* mRNA expression was similar when iMatrix-511 and iMatrix-221 were used as the coating (mean [standard deviation]: iMatrix-511, 1.00 [0.14]; iMatrix-221, 1.18 [0.18]; *P* = 0.06; *n* = 6) (Fig. 4E). The results further showed that expression of *Integrin β3* mRNA was significantly decreased when iMatrix-221 was used as a coating material compared with iMatrix-511 (mean [standard deviation]: iMatrix-511, 1.00 [0.30]; iMatrix-221, 0.08 [0.03]; *P* = 2.8E-05; *n* = 6) (Fig. 4F); expression of *Integrin β4* mRNA was decreased when iMatrix-221 was used as the coating material compared with iMatrix-511 (mean [standard deviation]: iMatrix-511, 1.00 [0.62]; iMatrix-221, 0.63 [0.42]; *P* = 0.28; *n* = 6) (Fig. 4G).

#### Adherens junctions

**Figure 5.**
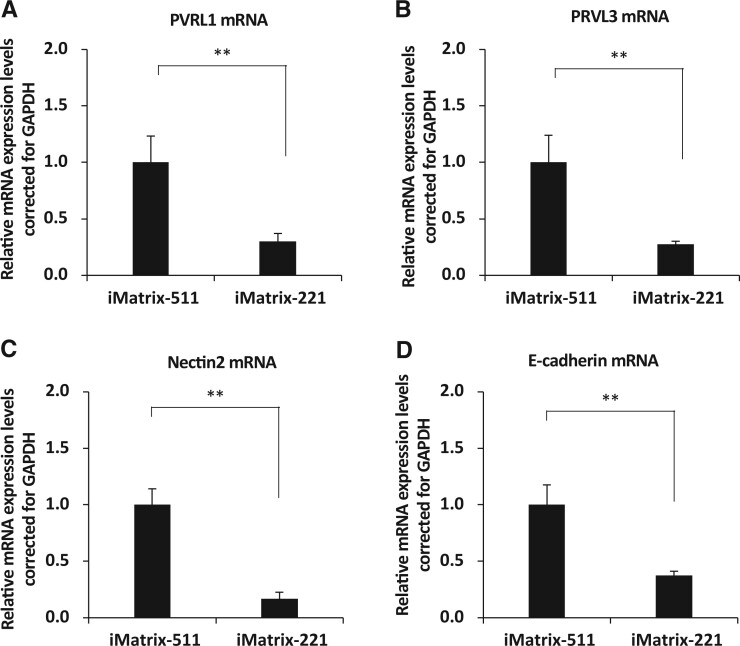
The evaluation of adherens junction. (**A**) *PVRL1*, (**B**) *PVRL3*, (**C**) *Nectin2* and (**D**) *E-cadherin* mRNA expression from hiPSCs on iMatrix511 or iMatrix-221 was measured by real-time PCR. Values indicate the relative value obtained by converting the calculated value of iMatrix511 to 1 (*n* = 3). The data are presented as the mean ± standard error. **P < 0.01.

PVRL1 (NECTIN1), PVRL3 (NECTIN3), Nectin2 and E-cadherin (Cadherin 1) are markers of adherens junctions [61, 62]. Analysis of PVRL1, PVRL3, Nectin2 and *E-cadherin* mRNA expression in hiPSCs (201B7 cell line) showed that *PVRL1* mRNA expression was significantly decreased when iMatrix-221 was used as the coating material compared with iMatrix-511 (mean [standard deviation]: iMatrix-511, 1.00 [0.23]; iMatrix-221, 0.30 [0.07]; *P* = 5.6E-05; *n* = 6) (Fig. 5A). *PVRL3* mRNA expression was significantly decreased when iMatrix-221 was used as the coating material compared with iMatrix-511 (mean [standard deviation]: iMatrix-511, 1.00 [0.24]; iMatrix-221, 0.28 [0.02]; *P* = 3.7E-05; *n* = 6) (Fig. 5B). *Nectin2* mRNA expression was significantly decreased when iMatrix-221 was used as the coating material compared with iMatrix-511 (mean [standard deviation]: iMatrix-511, 1.00 [0.14]; iMatrix-221, 0.17 [0.06]; *P* = 1.7E-07; *n* = 6) (Fig. 5C). *E-cadherin* mRNA expression was significantly decreased when iMatrix-221 was used as the coating material compared with iMatrix-511 (mean [standard deviation]: iMatrix-511, 1.00 [0.18]; iMatrix-221, 0.38 [0.03]; *P* = 1.1E-05; *n* = 6) (Fig. 5D).

Based on the results of the mRNA expression analyses of factors related to cell localization, we concluded that hiPSCs (201B7 cell line) cultured on coating material iMatrix-221 had a significantly reduced cell polarity compared to cells cultured on iMatrix-511.

### The analysis of cardiomyocytes differentiated from hiPSCs using iMatrix-511 and 221 coating

Krtolica *et al*. suggested that depolarization can affect the induction, proliferation and differentiation of human ES cells during embryoid body formation as depolarized human ES cells predominantly undergo haematoendothelial differentiation [35]. We therefore evaluated the potential utility of depolarization in clinical applications by inducing cardiomyocyte differentiation in hiPSCs cultured on iMatrix-511 and iMatrix-221.

**Figure 6.**
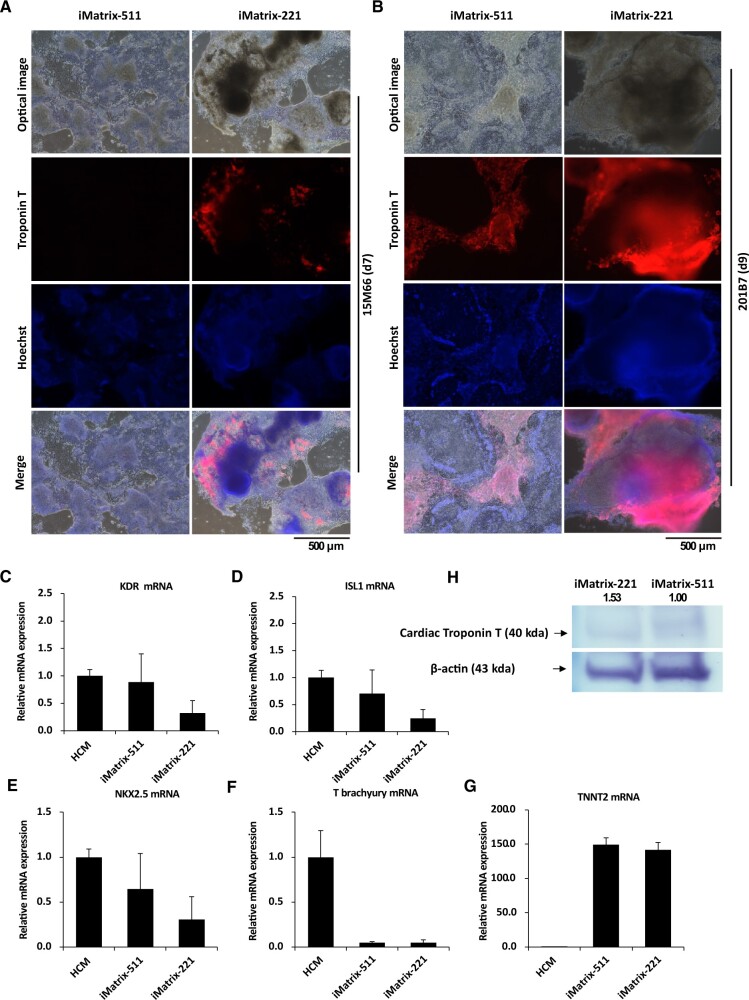
Effect of iMatrix-221 on the induction of differentiation into cardiomyocytes. (**A**) Cardiomyocyte marker expression at Day 7 after the start of induction of differentiation to cardiomyocytes in the hiPSC line 15M66. Optical microscope images are shown (top panels). The cells were then immunostained using Troponin T antibodies (second from the top) and Hoechst (third from the top). Bottom panels show merged images. Scale bar = 500 μm. (**B**) Cardiomyocytes on Day 9 after the start of differentiation induction from hiPSC line 201B7. Optical microscope images are shown (top panels). Cells were then immunostained using Troponin T antibodies (second from the top) and Hoechst (third from the top). Bottom panels show merged images. Scale bar = 500 μm. (**C**) *KDR*, (**D**) *ISL1*, (**E**) *NKX2.5*, (**F**) *T brachyury* and (**G**) *TNNT2* mRNA expression in hiPSCs on iMatrix511 or iMatrix-221 was measured by real-time PCR. Values indicate the relative value obtained by converting the calculated value of iMatrix511 to 1 (*n* = 3). The data are presented as the mean ± standard error. (**H**) Western blot assay image of cardiac Troponin T protein (top panel) and β-actin (bottom panel) bands detected by chemical colouration on the membrane using TMB substrate for HRP. The samples flowed into each lane (10 μl) of the membrane.

Human mononuclear cells isolated from blood samples are the most common cell type derived from clinical hiPSCs. Therefore, 15M66, a human mononuclear cell-derived hiPSC, was used to induce cardiomyocyte differentiation. Cardiomyocytes differentiated on iMatrix-221 showed the presence of Troponin T-positive cells on Day 7 of differentiation (Fig. 6A, second panels from the top, on the right). In contrast, cardiomyocytes differentiated on iMatrix-511 showed no troponin T-positive cells at Day 7 of differentiation (Fig. 6A, second panels from the top, on the left). Optical micrographs and movies of hiPSCs (15M66) were obtained on Day 6 after the induction of cardiomyocyte differentiation on iMatrix-221 (Supplementary Fig. S4A, top panels) (file name below the picture indicates the corresponding movie file name) and iMatrix-511 (Supplementary Fig. S4A, lower panels). Cardiomyocytes differentiation occurred earlier in cultures on iMatrix-221 and a larger tissue mass was formed compared to cultures on iMatrix-511. Since vascular endothelial cells and cardiomyocytes are analogous, our observations are consistent with a previous report [35].

To confirm the results from the 15M66 cells, we analysed cardiomyocyte differentiation using the 201B7 cell line. Fluorescence immunostaining images on Day 9 after differentiation induction were obtained. Cardiomyocytes differentiating on iMatrix-221 included troponin T-positive cells in a large tissue mass on Day 9 (Fig. 6B, second panel from the top, on the right). In contrast, cardiomyocytes differentiating on iMatrix-511 included planar troponin T-positive cells on Day 9 (Fig. 6B, second panel from the top, on the left). Optical micrographs and movies of hiPSCs (201B7) were obtained at Day 9 after induction of differentiation into cardiomyocytes for cultures on iMatrix-221 (Supplementary Fig. S4B, top panels) (file names below the pictures indicate the corresponding video file names) and on iMatrix-511 (Supplementary Fig. S4B, lower panels). We sought to determine whether the morphology of cardiomyocytes induced from iPSCs in a semi-floating culture on iMatrix-221 was induced by iMatrix-221-specific signals or simply depended on the low adhesiveness of iMatrix-221 compared to iMatrix-511. Cardiomyocytes were induced to differentiate from the 201B7 cell line in wells coated with iMatrix-511 alone or iMatrix-511 and iMatrix-221 together. On Day 14 after the induction of differentiation, beating cardiomyocytes were observed in wells coated with iMatrix-511 alone and in wells with iMatrix-511 and iMatrix-221 together. Furthermore, cardiomyocytes induced to differentiate in wells with iMatrix-511 alone showed a sheet-like tissue morphology with weak uplift, while those induced to differentiate in wells coated with iMatrix-511 and iMatrix-221 together showed high uplift and many areas of semi-floating tissue morphology (Supplementary Fig. S5A). These semi-floating tissues were observed on confluent cell sheets. These findings indicate that the semi-floating state of cardiomyocytes induced to differentiate on iMatrix-221 is not due to the low adhesiveness of iMatrix-221.

The differentiation of hiPSCs to cardiomyocytes is influenced by many substrate characteristics, including stiffness [63]. We investigated the possibility that on low-adhesion Laminin 221, cells are insensitive to the stiffness of the polystyrene substrate because they are not in contact with it, whereas, on Laminin 511, cells can sense the stiffness of the substrate because they are firmly attached to it. Six-well plates with well bottoms made of silicon materials of different hardness (0.2, 0.5, 2, 8, 16, 32 and 64 kPa) were used. Cells were analysed under an optical microscope on Day 5 after seeding. The adhesion of iPSCs to silicon was weak not only with an iMatrix-221 coating but also with an iMatrix-511 coating. Some adherent colonies were observed, but most were non-adherent EB-like cell masses (Supplementary Fig. S5B). However, in iMatrix-511-coated wells of 8 kPa, iPSCs showed semi-floating colonies. In addition, iMatrix-511-coated iPSCs showed clumps of adherent iPSCs on the bottom of the wells of 8 kPa. We used well plates with a range of hardness for the differentiation induction experiments, taking care not to aspirate floating iPSC colonies. An optical microscopy image obtained on Day 13 after induction of differentiation into cardiomyocytes is shown in Supplementary Fig. S5C. Cardiomyocytes induced to differentiate in wells of different hardness showed no significant morphological differences on either iMatrix-511 or iMatrix-221; in all cultures, tissue morphology showed a slightly raised tissue mass and surrounding fibroblast-like cell sheets. However, the size of the adherent tissue mass occupying the well base was greatest in the 8 kPa well (Supplementary Fig. S5C).

mRNA expression in cardiomyocytes at Day 14 was examined by real-time PCR for the following target genes: NK2 homeobox 5 (NKX2.5), a marker of cardiac progenitor cells; Islet1 (ISL1), which is expressed in the secondary heart field of the visceral mesoderm and is necessary for the proliferation and maintenance of cardiac progenitor cells; T brachyury, which is essential for cell differentiation into the mesodermal lineage; and TNNT2, a protein that is part of the myofibrils of cardiac muscle. Expression of *NKX2.5* and *ISL1* mRNAs was not characterized by any particular trend in cardiomyocytes induced to differentiate on iMatrix-511 in 0.2–64 kPa wells. In contrast, expression of *NKX2.5* mRNA (Supplementary Fig. S6A) and *ISL1* mRNA (Supplementary Fig. S6B) tended to be higher in cardiomyocytes induced to differentiate with iMatrix-221 in harder wells than on softer wells. Expression of *T brachyury* mRNA tended to be higher in cardiomyocytes induced to differentiate on iMatrix-511 in softer wells. In contrast, in cardiomyocytes induced to differentiate on iMatrix-221, expression of *T brachyury* mRNA tended to be lower than that in cardiomyocytes induced to differentiate on iMatrix-511 as a whole (Supplementary Fig. S6C). Expression of *TNNT2* mRNA showed a trend towards increased expression in 8 and 16 kPa wells in cardiomyocytes induced to differentiate on iMatrix-511. In contrast, *TNNT2* mRNA expression in cardiomyocytes differentiated on iMatrix-221 was low in 0.2–32 kPa wells but tended to be higher in 64 kPa wells (Supplementary Fig. S6D). These results show that not only silicon hardness but also the differences between iMatrix-511 and iMatrix-221 coatings affected the induction of differentiation of iPSCs into cardiomyocytes. Overall, the iMatrix-221 coating tended to be more effective in inducing differentiation and maturation into cardiomyocytes, even on hard wells, than the iMatrix-511 coating. In conclusion, iMatrix-221-coated semi-suspended cells are less sensitive to the stiffness of the well bottom and are as effective in inducing differentiation into cardiomyocytes as if they were on a soft well bottom.

The results of an mRNA expression analysis on Day 9 after induction of cardiomyocyte differentiation using hiPSCs (201B7 cell line) to assess differentiation maturity are shown. The kinase insert domain receptor (KDR) [64], ISL1 [65, 66] and Nkx2.5 [67, 68] are markers of cardiovascular progenitor cells. *KDR*, *ISL1* and *NKX2.5* mRNA expression on Day 9 after induction of differentiation of hiPSCs (201B7 cell line) into cardiomyocytes did not differ significantly between iMatrix-511 and iMatrix-221. Human cardiomyocytes (HCMs) were used as a control, and their mRNA expression was set as 1. The data from the HCMs were not used for the significance test.

*KDR* (Fig. 6C), *ISL1* (Fig. 6D) and *NKX2.5* (Fig. 6E) mRNA expression did not differ significantly between iMatrix-511 and iMatrix-221. However, the levels of expression of markers of cardiovascular progenitor cells in cardiomyocytes differentiated on iMatrix-221 were lower than those of that differentiated on iMatrix-511. On Day 9 after induction of hiPSC (201B7 cell line) differentiation into cardiomyocytes, the level of mRNA expression of the mesoderm progenitor marker T (Brachyury) [69] was similar between iMatrix-511 and iMatrix-221, and both had a lower level of expression than control HCMs (Fig. 6F). The levels of mRNA expression of troponin T (TNNT2) were similar in iMatrix-511 and iMatrix-221 cultures; expression of TNNT2 was higher than that of control HCMs (Fig. 6G). The levels of TNNT2 protein on Day 9 after induction of hiPSC (201B7 cell line) differentiation into cardiomyocytes was identical in iMatrix-511 and iMatrix-221 cultures when corrected for the expression of β-actin (Fig. 6H).

Based on the analysis of fluorescence immunostaining images (Fig. 6A: TNNT2, second panels from the top) and movies (Supplementary Fig. S4A, top panels), the induction of cardiomyocytes from hiPSCs on iMatrix-221 shortened the differentiation period, and the induced differentiation tended to result in the formation of tissue mass. In contrast, no notable difference between iMatrix-511 and iMatrix-221 coating was observed in the final maturation stage of differentiation, based on the mRNA and uncorrected Western blot films (Supplementary Fig. S7, top panel: TNNT2, lower panel: β-actin).

## Discussion

Our mission is to develop an industrialized technology capable of providing therapeutic cells for clinical use by applying basic research using iPSCs. Industrialization of the processes for bulk production of next-generation therapeutic cells, such as automated clinical iPSC production, passaging of transduced cells without colony picking after exposure to an initializing gene expression vector, is essential for clinical iPSC production. At present, iPSCs produced in bulk on Laminin 511 are contaminated with iPS-like cells. Furthermore, some cells that acquire proliferative ability have characteristics more similar to somatic cells than iPS-like cells. These cells show the ability to adhere not only to Laminin 511 but also to the bottom surface of the uncoated culture vessels (Supplementary Figs S2A and S6A and B). Therefore, there is a need for vectors for iPSC establishment that can generate a single-cell population in bulk without colony picking; currently, however, such vectors seem next to impossible to obtain.

Against this background, we investigated the potential utility of Laminin 221 in the present study. Laminin 211/221 is present in skeletal muscle; abnormality of these laminins causes congenital muscular dystrophy. Analyses of multiple laminins showed that Laminin 511 is suitable as a coating material for mouse ES cell culture, while Laminins 332, 111 and 411 are not suitable for this purpose [70]. Subsequently, Laminin 511 was also shown to be a suitable coating material for human ES cells [71]. Laminin 511 E8 is thus at present the most widely used coating material for clinical iPSC culture [17]. Laminin 211 has also been used occasionally as a culture coating material for culturing human ES cells [72].

In comparison with Matrigel, the classical coating material, Laminin 211 has a similar function in inducing differentiation of human foetal cardiac mesenchymal stem cells into cardiomyocytes [73]. The basis of this property is that Laminin 211/221 is a cell adhesion molecule that is strongly expressed in the basement membrane of skeletal muscle [19]. A detailed examination of the distribution of laminins in the skeletal muscle basement membrane showed that Laminin 211/221 is the most abundant, with Laminin 411/421 and Laminin 511/521 also reported to be present [22]. Recently, Laminin 221 was identified as the most highly expressed cardiac laminin. With regard to the induction of HCM differentiation, the human ES cell line H1 cultured with Laminin 521 + Laminin 221 coating (compared to Laminin 521 only, Laminin 521 + Laminin 211 and vitronectin) had the largest increase in expression of the cardiac markers TNNT2 and MYH1 [74]. It has also been reported that Laminin 511/521 coating promotes maturation of thicker myofibres in the myocardium [75].

hPSCs mainly express integrin α6β1 [76, 77], which has a particularly high affinity for Laminin 511 but a low affinity for Laminin 211 [78, 79]. Laminin 511 binds tightly to integrin α6β1 [18, 80], whereas Laminin 211/221 binds to integrin α7β1 [18, 81]. Laminin 511 activates the α6β1 integrin-Fyn-RhoA-ROCK signalling pathway via integrins in iPSCs [58]. In brief, activation of the α6β1 integrin-Fyn-RhoA-ROCK signalling pathway accelerates phosphorylation of Rac1, strongly upregulates actin-related cytoskeletal formation, and suppresses the Notch signalling pathway [82]. Thus, use of Laminin 221 protects this Notch signalling pathway, and disables the α6β1 integrin-Fyn-RhoA-ROCK signalling pathway. Activation of Rac1 by laminin and dynamic changes in the actin backbone are presumed to be responsible for the development of cell polarity [83]. We recently reported that iPSCs express *ADGRG6* mRNA (GPR126, VIGR, DREG) [84], an adhesion G protein-coupled receptor known to be a receptor for Laminin 211 [85, 86]. During cardiomyocyte development, ADGRG6 has been reported to be an essential factor for heart valve development [87, 88]. Because of its value, cardiac surgeons have proposed that 3D-Engineered Cardiac Tissues, an iPS cell-derived cardiac myocardial sheet used for transplantation, is produced in cultures coated with fibrinogen and Laminin 221. One prerequisite for cardiomyocytes that show therapeutic efficacy *in vivo* is having high viability and survival under 3D culture conditions [21]. Laminin 211/221 exhibits two characteristics: cardioprotective effect and low adhesiveness to iPSCs. In the present study, we found that iPSCs can be established on Laminin 211/221 and demonstrated that cardiomyocytes could be established from iPSCs using only Laminin 211/221.iPSC establishment efficiency was investigated here. The SeV vector for iPSC establishment used in this experiment is a research product that is not approved for clinical iPSC establishment. Therefore, we also used a different vector, CytoTune^®^-iPSC 2.0, and followed the manufacturer’s protocol; this vector is in current use for clinical iPSC establishment. We found that iMatrix-221, iMatrix-411 and vitronectin had comparable iPSC establishment efficiencies to iMatrix-511 when used as the coating for establishing hiPSCs using mononuclear cells isolated from patient-derived blood (Fig. 2D). iMatrix-221, iMatrix-411 and vitronectin could be used in accordance with existing clinical iPSC establishment protocols in addition to iMatrix-511 to ensure iPSC establishment efficiency. Next, we surveyed cultures at each passage to determine the level of residual SeV following initial transfection to establish iPSCs. SeV vectors are necessary for cell initialization but must thereafter be rapidly shed from the cells; iPSCs containing SeV vectors that continue to express the initialization factor intracellularly cannot initiate the differentiation induction step. Therefore, we used the SRV™ iPSC-2 Vector, which is expected to exit cells early, and exposed cells to the vector for 2 h. The shortest time to obtain hiPSCs free from SeV vector was three passages for iMatrix-411 and iMatrix-221 and four passages for vitronectin (Fig. 2A and B). The number of days required for each round of passaging is ∼7 days; the length of time between passages is important as it is the absolute rate-limiting factor for shipping time in the clinical cell manufacturing process. Since hiPSCs cultured on iMatrix-221 are slow-growing (Fig. 2C), it is desirable to set the cell seeding density to 5 × 10^4^ cells/well (six-well plates); this is 4–5 times higher than that for iMatrix-511 (Fig. 3A). We also found that colonies of hiPSCs cultured on iMatrix-221 showed the characteristics of semi-floating culture in which adherent cells and floating cells are mixed in a single colony (Fig. 3A). In addition, the morphology of the colonies was not ES cell-like, typical hiPSC colony form, which is a quality evaluation standard for clinical iPSCs. hiPSCs cultured on iMatrix-221 were characterized by a significant increase in β-actin (Fig. 3B), α-E catenin (Fig. 3G, left), β-catenin (Fig. 3B, right), and Integrin α1 (Fig. 4A), which are major factors in cell polarity associated with epithelial cells. However, these hiPSCs also showed a significant decrease in SCRIB (Fig. 3C), ZO-1 (Fig. 3D), CRB3 (Fig. 3E), PARD3 (Fig. 3F), Occludin (Fig. 3H), Integrin α6 (Fig. 3C), Integrin β3 (Fig. 4F), PVRL1 (Fig. 5A), PVRL3 (Fig. 5B), Nectin2 (Fig. 5C) and E-cadherin (Fig. 5D), other factors associated with cell polarity in epithelial cells. These large-scale changes in the expression of factors associated with cell polarity may have caused the changes in morphology of hiPSCs [89–91] from ES cell-like to peninsular-like. Cardiomyocytes that differentiated on iMatrix-221 were precocious (Fig. 6A) and had normal maturation completion (Fig. 6B–H). In contrast, cardiomyocytes that differentiated on iMatrix-221 showed 3D tissue formation with part of the tissue mass free from the surface of the culture dish (Supplementary Fig. S4A and B), resulting in semi-3D cardiomyocytes. Previous reports have shown that iMatrix-221 induces differentiation of neural crest cells [92], suggesting that iMatrix-221 may promote 3D tissue induction not only in neural organs and cardiac muscle but also in a wider range of other organ types.

In summary, the use of iMatrix-221 coating for the establishment, culture, passaging and differentiation of hiPSCs into cardiomyocytes allowed us to perform semi-3D culture with reduced cell polarity. These findings suggest that this protocol could contribute to the development of a new phase of clinical application research as it provides a culture method that bridges the existing 2D and 3D differentiation induction methods that are currently being actively applied in the differentiation-induction step of clinical hiPSC generation.

## Supplementary data

Supplementary data are available at *REGBIO* online.

## Supplementary Material

rbac060_Supplementary_Data

## Data Availability

In this paper, the video with the following file name is provided as available data: movie: 20211130-hart-d9-201B7-iMat221-1.avi
