# Supplementary figures and images for: Semi-3D cultures using Laminin 221 as a coating material for human induced pluripotent stem cells
Source: Regen Biomater. 2022 Sep 5;9:rbac060. doi: 10.1093/rb/rbac060 (PMC9514851; doi:10.1093/rb/rbac060)

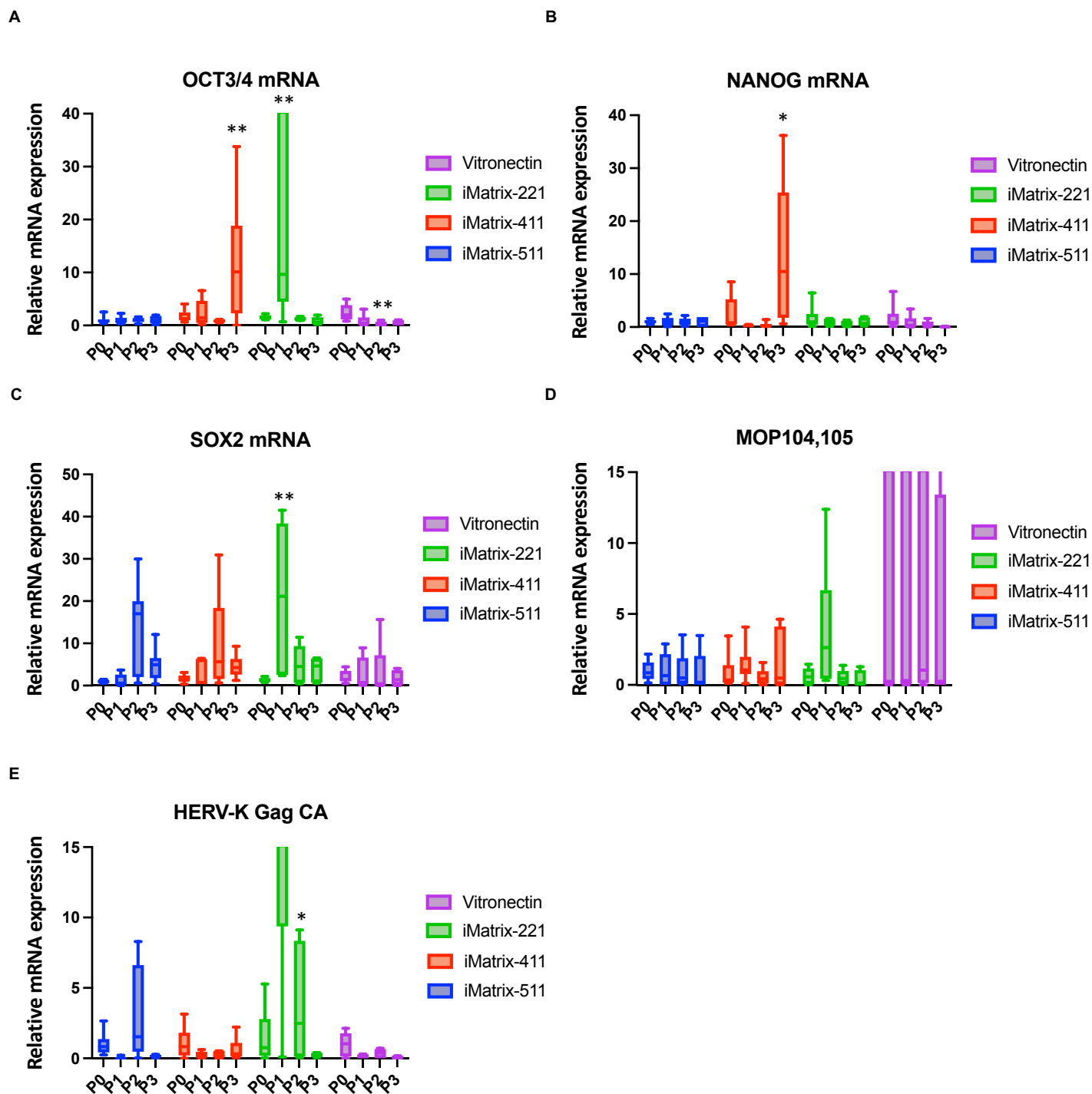

A

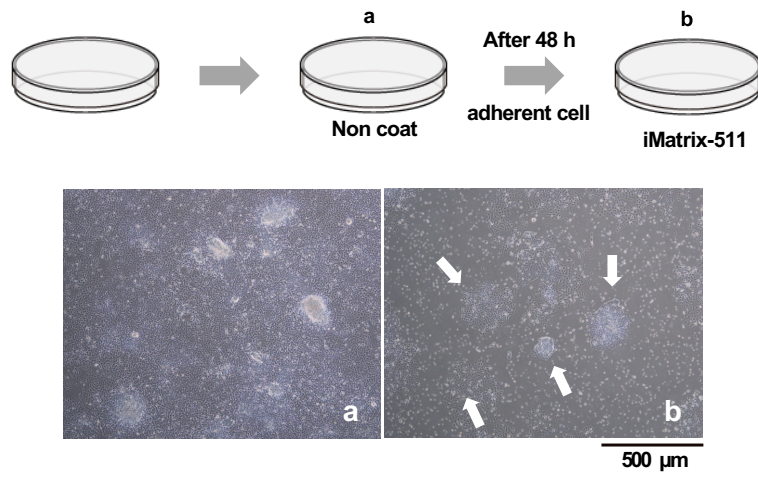

B

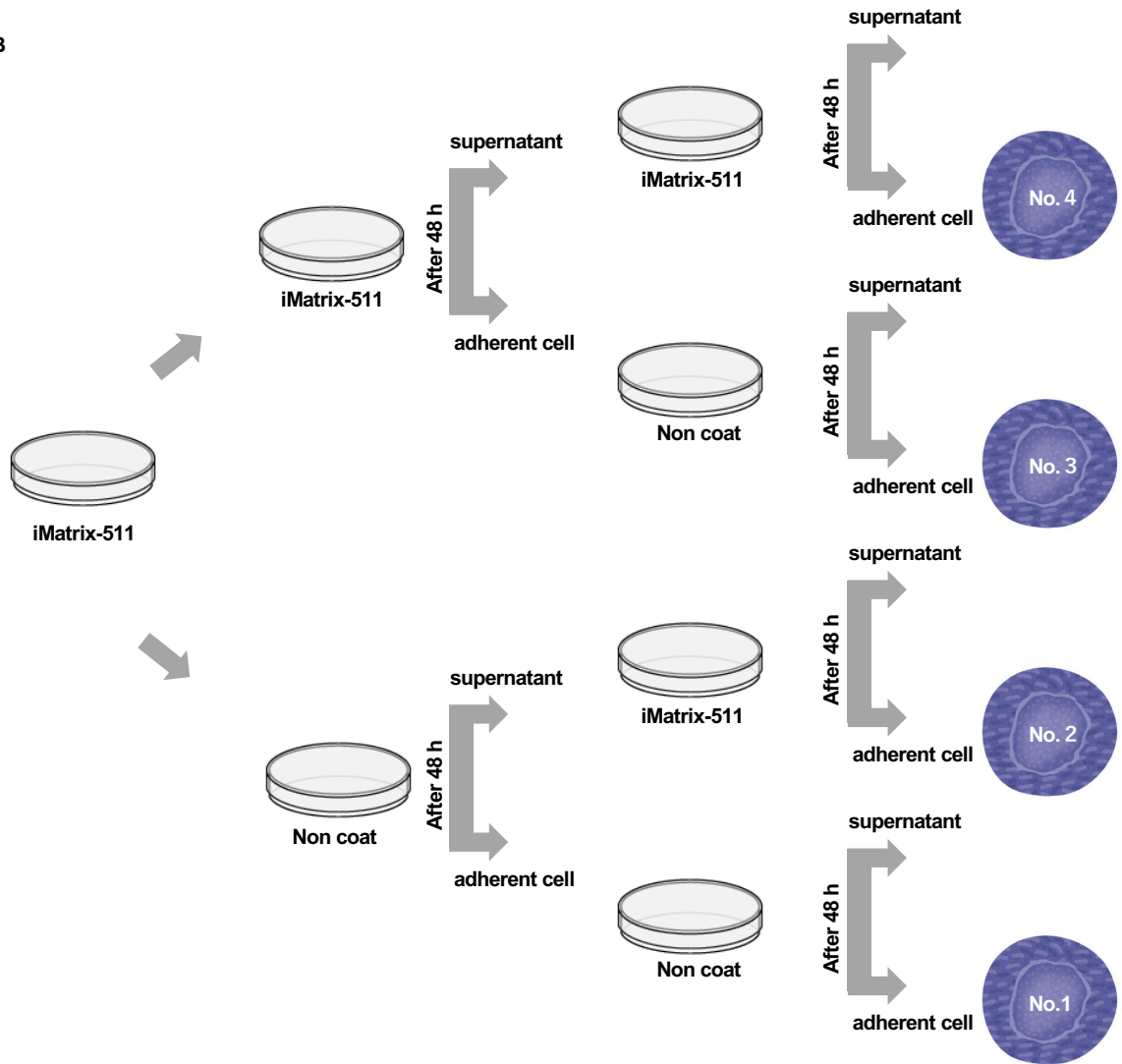

A

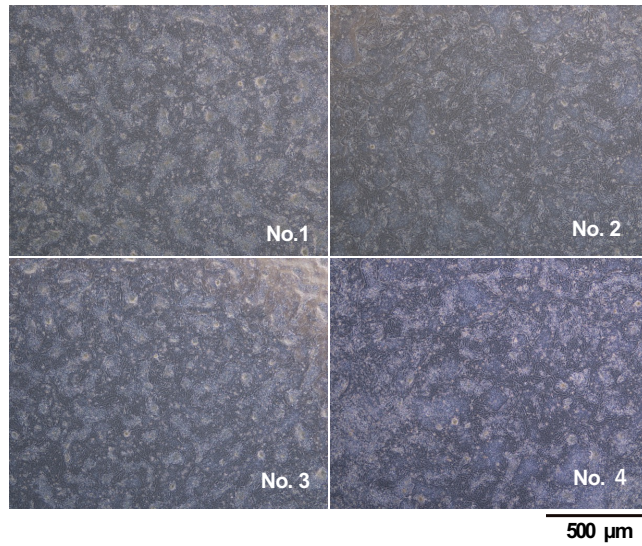

B

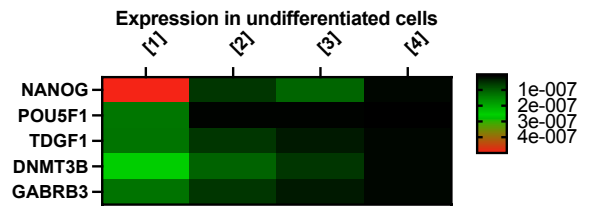

C

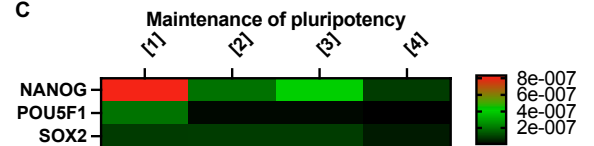

E

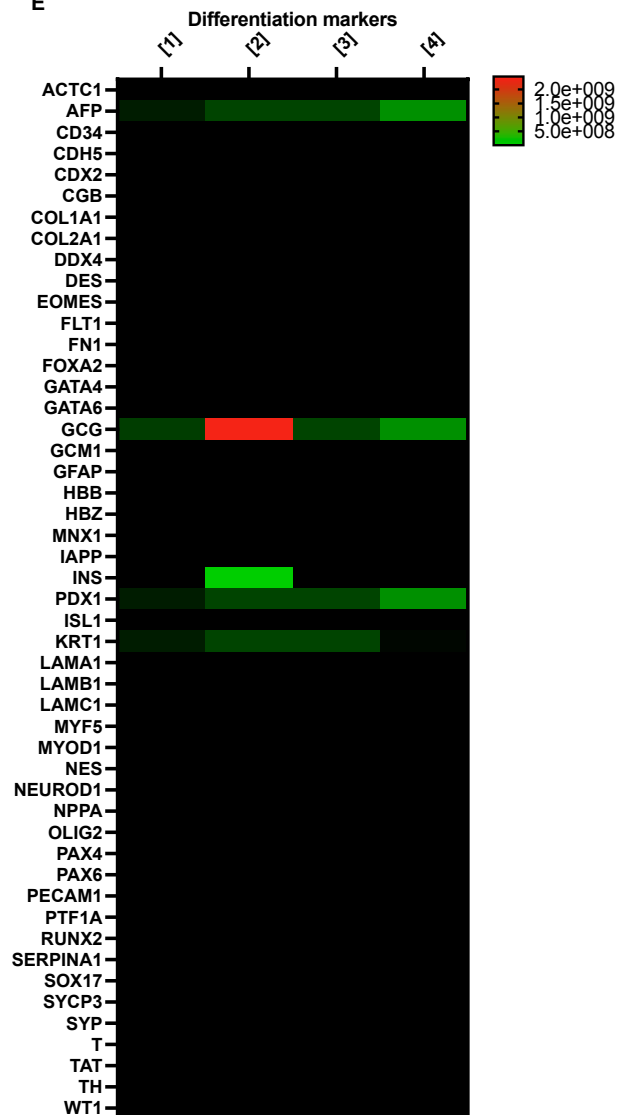

D

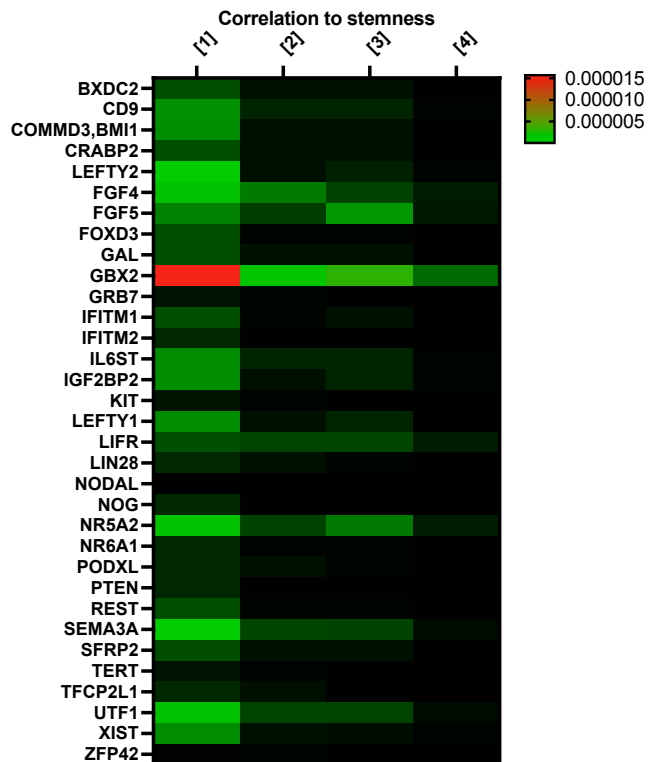

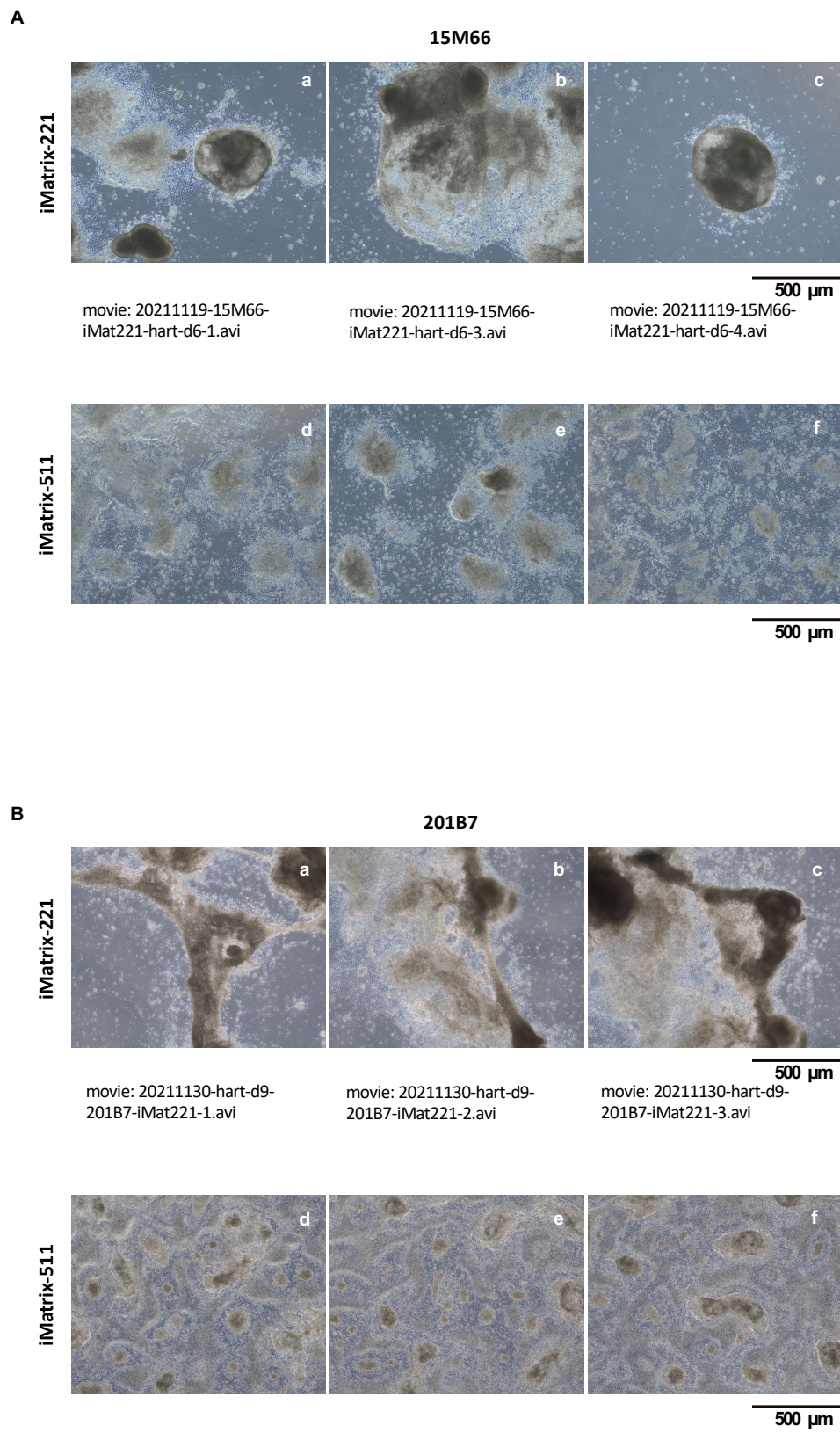

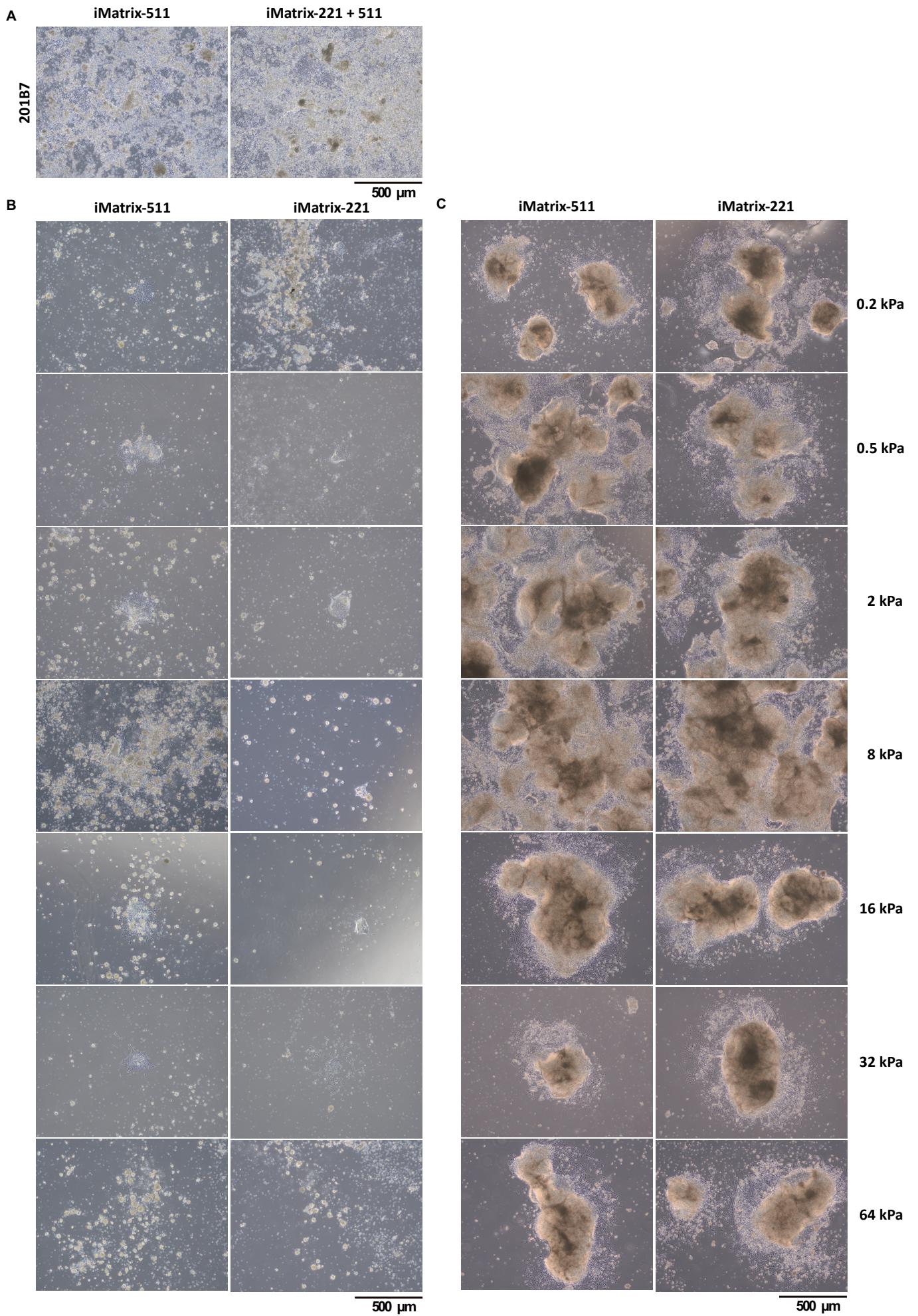

Nakashima et al. Supplemental Figure 5

A

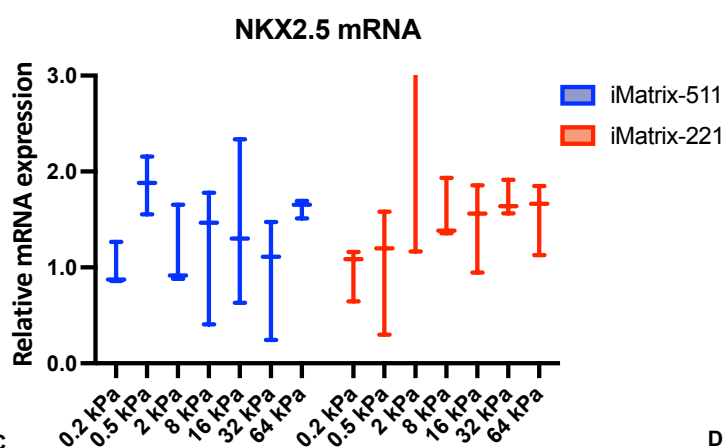

B

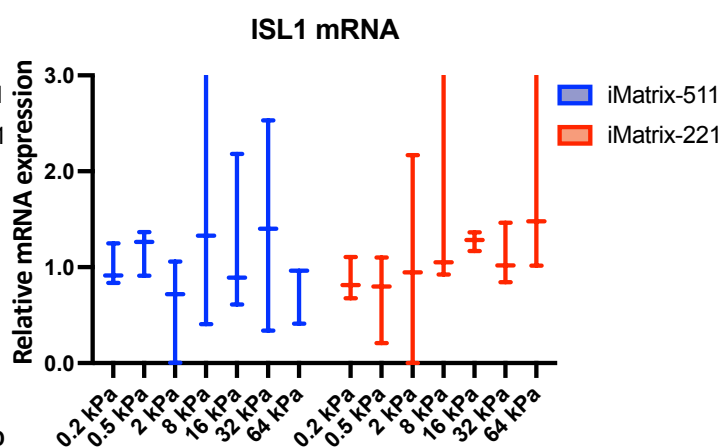

C

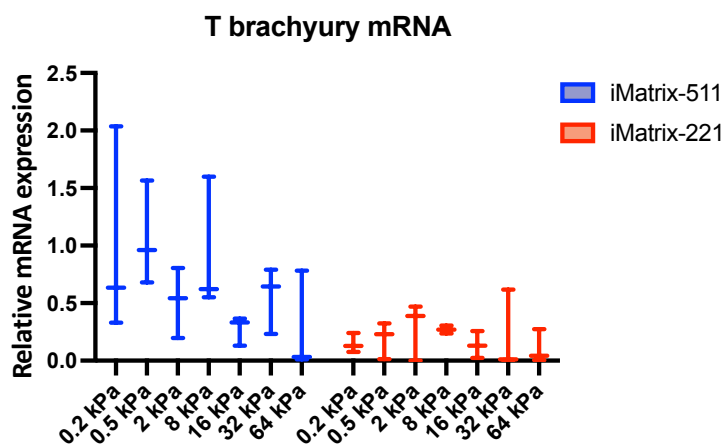

D

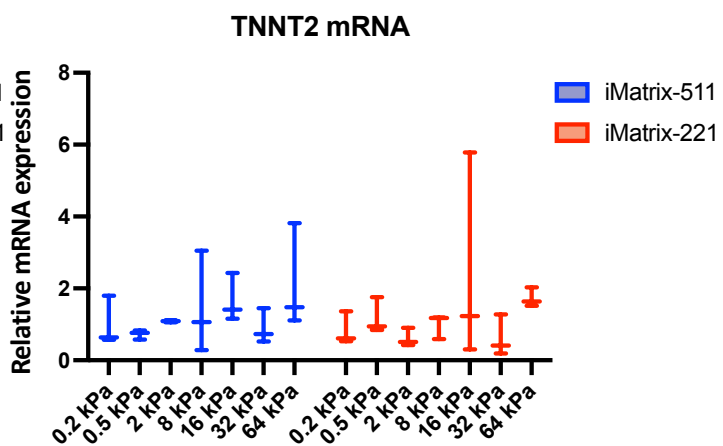

Uncorrected Western blot films

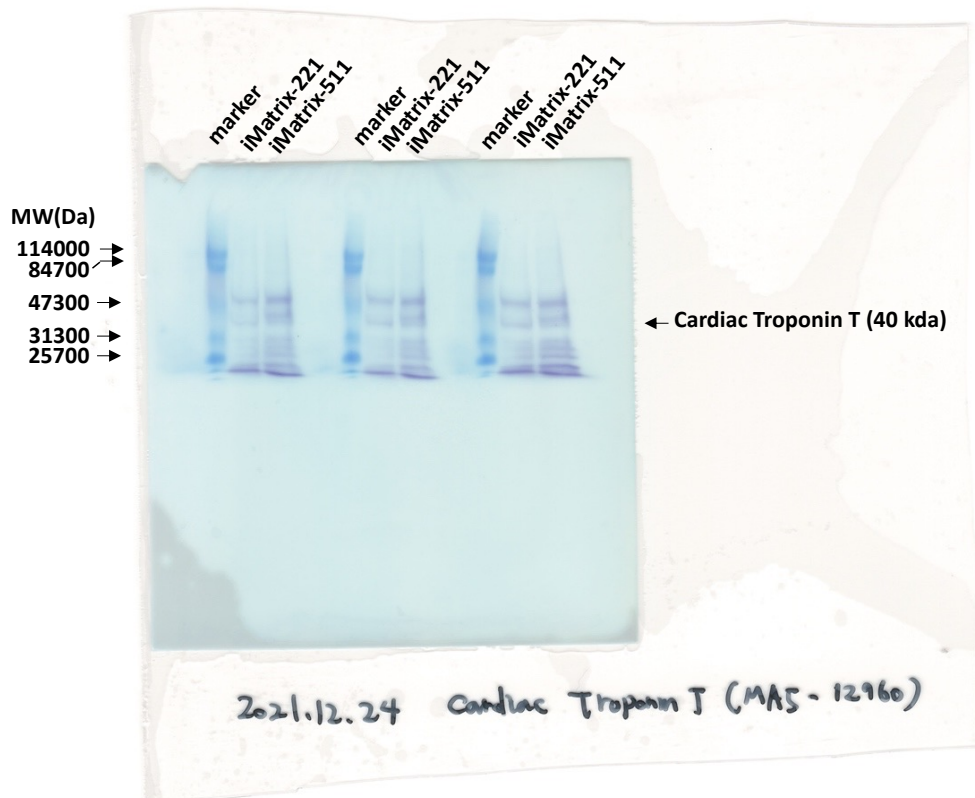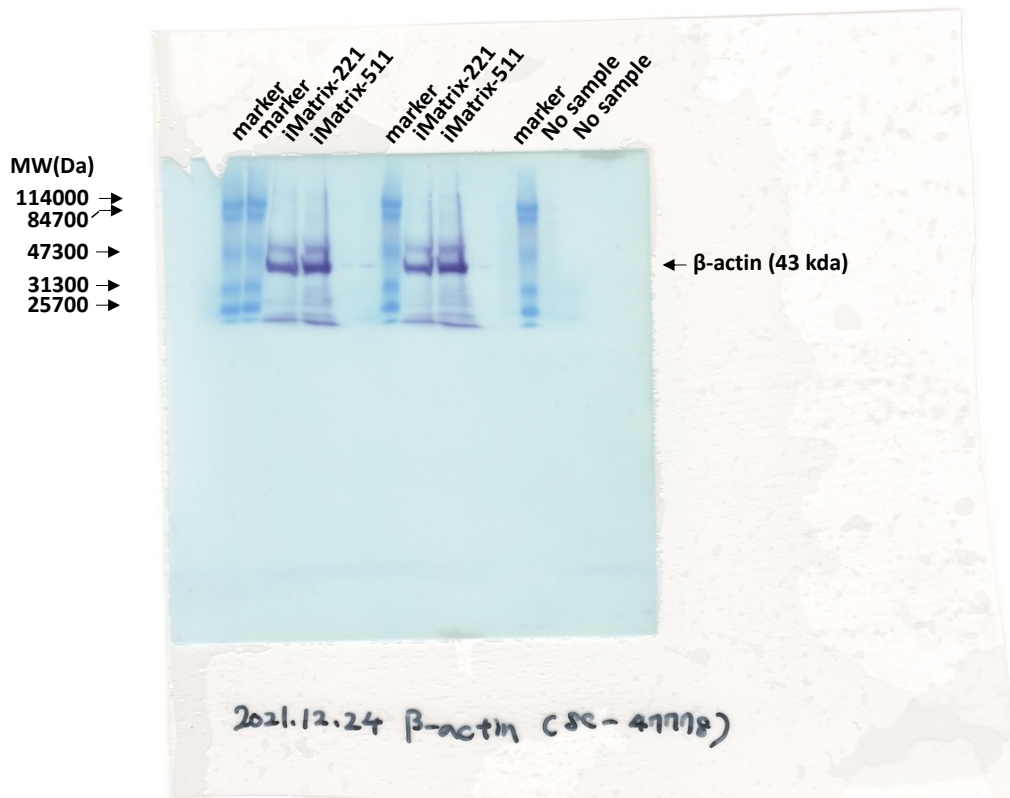

Supplement: rbac060_Supplementary_Data [file rbac060_supplementary_data.pdf]
